# ABA-Type Thermoplastic Elastomers: From Styrenic and Acrylic Systems to Emerging Bio-Based Materials

**DOI:** 10.3390/ma19163532

**Published:** 2026-08-20

**Authors:** Aniello Vittore, Orlando Santoro, Lorella Izzo

**Affiliations:** Laboratory of Polymer Chemistry and Sustainable Catalysis, Department of Biotechnology and Life Sciences (DBSV), University of Insubria, 21100 Varese, Italy

**Keywords:** thermoplastic elastomers, ABA triblock copolymers, styrenic block copolymers (SBCs), acrylic TPEs, bio-based TPEs

## Abstract

The combined elasticity, processability, and recyclability of thermoplastic elastomers (TPEs) has enabled their widespread adoption across diverse industrial sectors. In particular, TPEs have emerged as attractive alternatives to chemically crosslinked elastomers, contributing to extended product lifetimes and reduced waste generation. Their performance arises from the presence of physical, reversible crosslinks, which allow the integration of elastomeric behaviour with thermoplastic reprocessability. This review provides an overview of recent advances in ABA thermoplastic elastomer (TPE) design, focusing on three major classes: styrenic block copolymers, acrylic-based TPEs, and emerging bio-based systems, with particular emphasis on the latter. Special attention is given to structure–property relationships and the influence of molecular architecture on thermomechanical behaviour. Styrenic block copolymers remain the most established class, offering well-defined phase-separated morphologies and tuneable mechanical properties. Acrylic-based TPEs have attracted increasing interest owing to their superior thermal and oxidative stability and versatile molecular design. We also discuss recent progress in bio-based TPEs derived from renewable resources, which aim to reduce reliance on fossil feedstocks without compromising performance. Finally, we examine current challenges and future perspectives, highlighting the need for sustainable synthetic strategies and advanced TPEs with lower environmental impact.

## 1. Introduction

**Figure 1:**
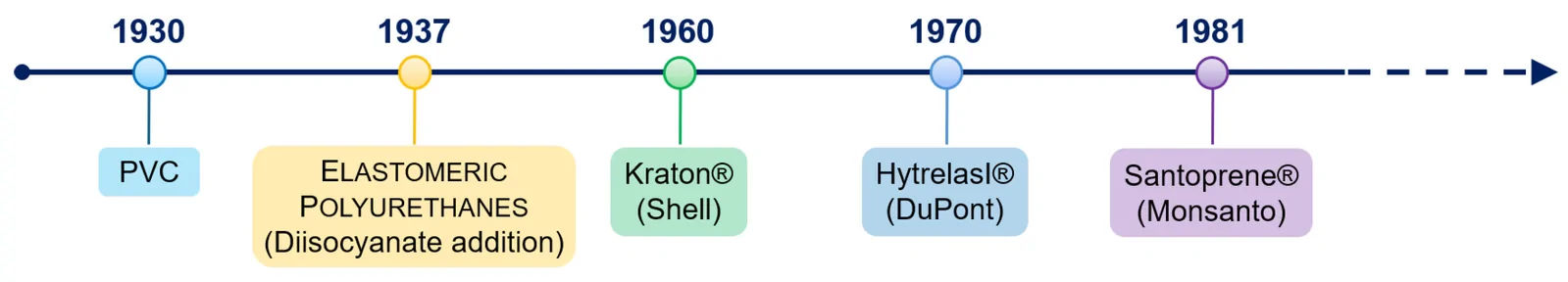
Thermoplastic elastomer (TPE) development timeline [1,2,3,4,5].

Interest in flexible plastics started around the 1930s with the plasticisation of poly(vinyl chloride) (PVC), which had a rubber-like appearance [1]. In 1937, a breakthrough occurred with the discovery of the diisocyanate polyaddition reaction, used to produce polyurethane fibres and, later, elastomeric polyurethanes. From the 1950s, DuPont developed linear copolyester fibres and, in the 1970s, commercialised this copolyester as a thermoplastic elastomer under the name HytrelasI^®^. In the 1960s, Shell industrialised styrene-diene block copolymers, marketed as Kraton^®^, and Monsanto started to develop thermoplastic vulcanisates, introduced in 1981 on the market as Santoprene^®^. Later in the same decade, polyamide-based thermoplastic elastomers were introduced [2,3,4]. In the last decades, there has been considerable progress in the field of TPEs, and many more companies have developed new products [5] (Figure 1).

The global TPE market size was valued at USD 32.65 billion in 2025 and is projected to grow from USD 34.58 billion in 2026 to USD 54.69 billion by 2034, at a Compound Annual Growth Rate of 5.90% [6]. The rising demand for flexible and durable materials such as TPEs in various industries, for example, automotive, construction, and consumer goods, drives the market’s growth worldwide. TPEs are also widely used in other fields, including wire insulation, footwear, adhesives, food packaging, polymer blending, and mainly in automotive applications [7].

**Figure 2:**
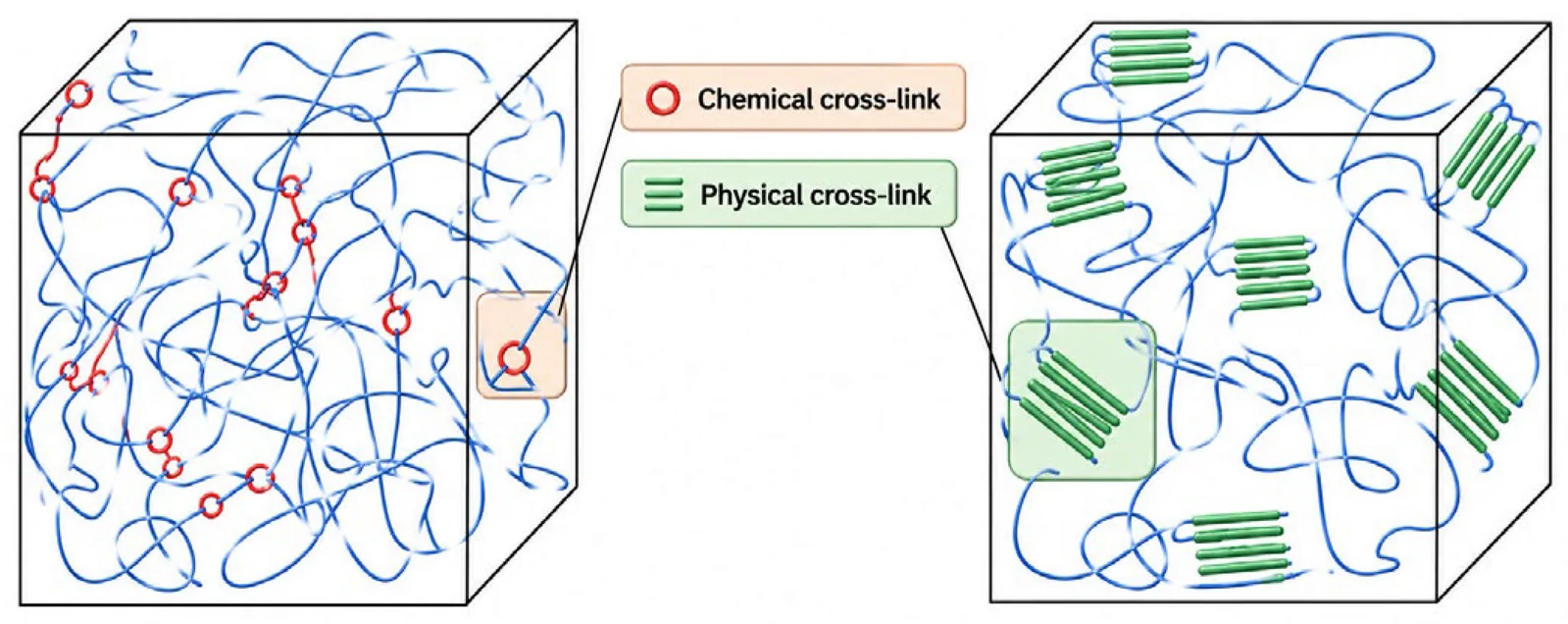
Differences between conventional rubbers and TPEs in terms of crosslink network. (The conceptual image was adapted from Ref. [9] and generated using OpenAI’s GPT-5.6 Luna model. The authors have reviewed and edited the output and take full responsibility for the content of this publication).

More often, TPEs are chosen for products over rubber because of their flexibility, versatility, and recyclability [8]; indeed, TPEs can combine the processing advantages of thermoplastics with the performance properties of elastomers. Consequently, they are relatively easy to process using thermoplastic processing methods such as extrusion and injection moulding, without requiring the vulcanisation process necessary for rubber materials (Figure 2, AI-generated) [9].

The unique combination of thermoplastic and elastomeric properties of TPEs arises from reversible networking as a result of physical crosslinks connecting long flexible polymer chains, allowing for large, reversible deformation and the preservation of the material’s strength. These reversible physical crosslinks enable processes such as melting or dissolution in appropriate solvents, unlike conventional rubbers, which, being thermoset materials, consist of chemical crosslinks that form an irreversible network. Compared to the vulcanised rubbers, the physical crosslinking of TPEs allows material recycling and reduces processing complexity and production costs, since a lower amount of energy is required to achieve control of product quality. On the other hand, this reversibility can also negatively affect some properties, like tolerance to solvents or the resistance to deformation at high temperatures, for which conventional thermoset rubbers normally have better performance. Nevertheless, the balance between their recyclability, cost efficiency, and versatile properties ensures their continued relevance and growth in the materials market.

In light of these premises and the increasing interest in these materials driven by their advantages over classic thermosets (particularly in terms of sustainability), this review aims at providing a comprehensive overview of recent advances in TPE design, focusing on three major classes, namely styrenic block copolymers (SBCs), acrylic-based TPEs, and emerging bio-based systems, sharing the same molecular architecture. Special emphasis is placed on structure–property relationships and the role of molecular architecture in governing thermomechanical behaviour. We report on how styrenic block copolymers represent the most established class, offering well-defined phase-separated morphologies and tuneable mechanical properties, while acrylic-based TPEs have gained increasing attention due to their enhanced thermal and oxidative stability, as well as their versatility in molecular design. Finally, we discuss the significant efforts that have been recently devoted to developing bio-based TPEs derived from renewable resources to reduce the reliance on fossil feedstocks without compromising performance. Current challenges and future perspectives are evaluated, highlighting the need for sustainable synthetic approaches and the design of more advanced TPEs with reduced environmental impact. Although recent reviews have individually discussed TPE families, advanced macromolecular architectures, and bio-based materials [10,11], a comprehensive comparison linking fossil-based SBCs, acrylic ABA systems, and the rapidly expanding family of renewable TPEs through common structure–property relationships is still lacking. This review aims to bridge these areas by critically comparing molecular design strategies focusing on the most prevalent structure, namely ABA.

## 2. Classification of TPEs

### 2.1. TPEs’ Chemical Composition

**Figure 3:**
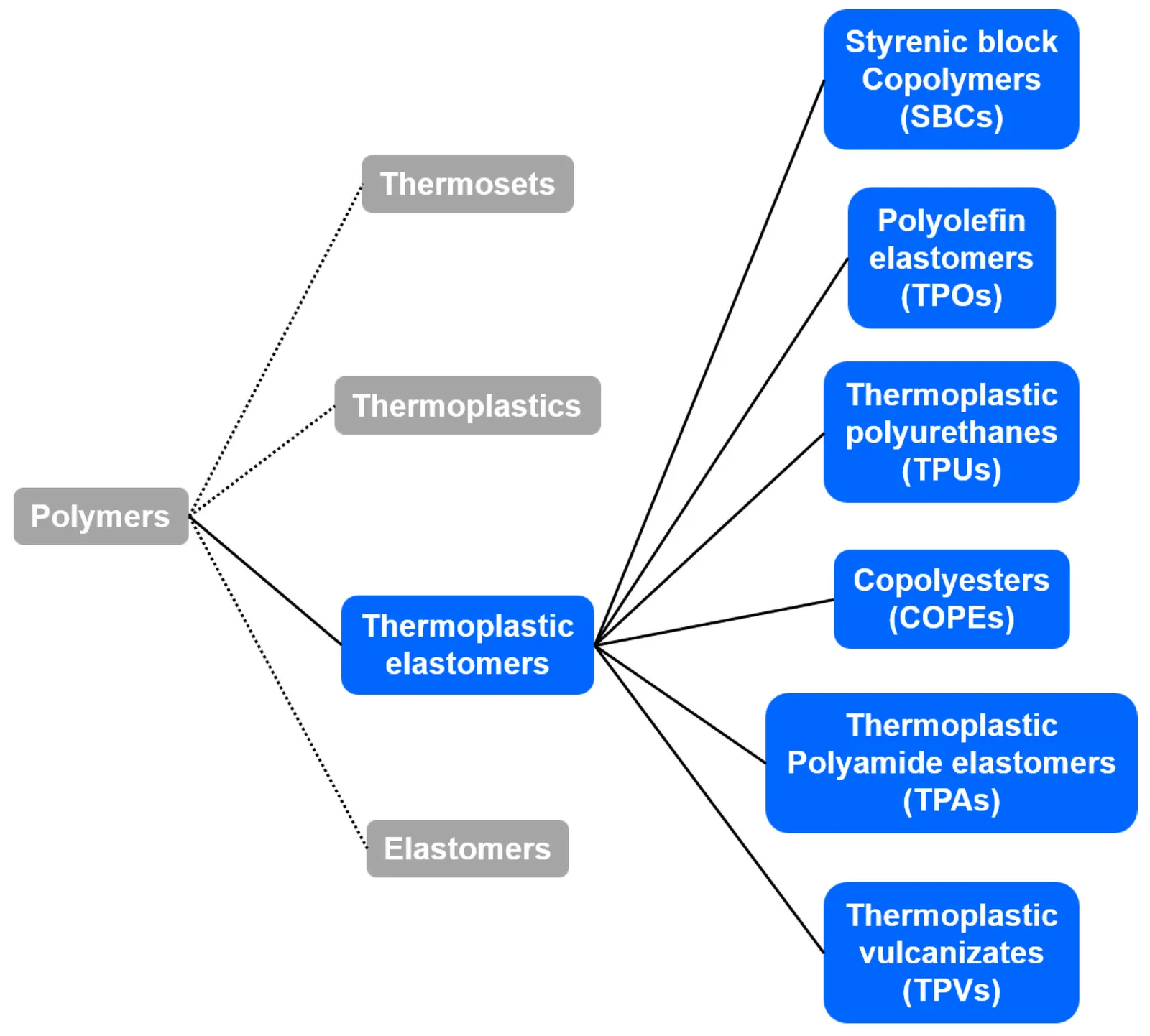
Main classification of polymers and thermoplastic elastomers classes.

TPEs are classified based on their main structure that includes styrenic block copolymers, polymer blends obtained by dynamic vulcanisation (TPVs), polyolefin-based thermoplastic elastomers (TPOs), thermoplastic polyurethane elastomers (TPUs), polyamide-based thermoplastic elastomers (COPA), and polyether ester elastomers (COPE) (Figure 3) [12].

Even though different chemical structures can form thermoplastic elastomers, they share a common multiblock network that leads to polymer phase separation. In some cases, these phases are not chemically connected and exist as blends, but more often, they are linked as block or grafted copolymers. To guarantee the fundamental TPEs’ properties, like thermoplastic processability and elastomeric behaviour, the presence of at least one elastomeric phase, having a glass transition temperature (*T*_g_) below room temperature (RT), and one hard phase (*T*_g_ or melting temperature-*T*_m_ > RT) are required. While both phases are essential to contribute to physical and mechanical characteristics, the hard phase is mostly responsible for the thermoplastic character and plastic properties like tensile strength, tear strength, resistance to chemicals, and adhesion properties. In contrast, the elastomeric properties, like hardness, flexibility, and compression set, are ascribed to the soft amorphous phase.

### 2.2. TPEs’ Architecture

**Figure 4:**
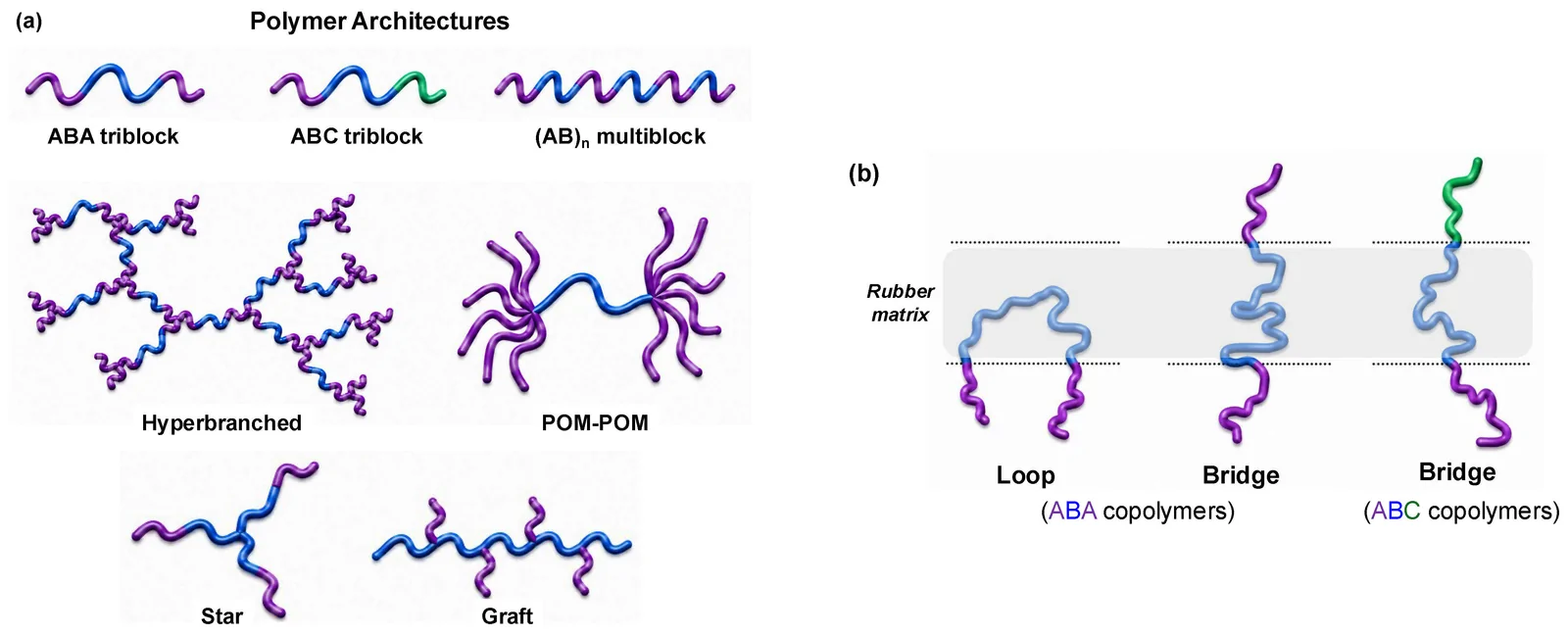
Polymer architectures of TPEs (**a**). Possible polymer chain conformations for ABA and ABC TPE copolymers (**b**). (The conceptual image was adapted from Ref. [8] and generated using OpenAI’s GPT-5.6 Luna model. The authors have reviewed and edited the output and take full responsibility for the content of this publication).

Macromolecular architectures of TPEs can vary widely, ranging from linear to graft and star-like structures. All these architectures must fulfil one fundamental requirement: ensuring the formation of crosslink junctions between the elastomer and glassy components. For example, TPEs can be formed by linear block copolymers; most feature triblock or multiblock structures like ABA, ABC, or (AB)_n_, where B is the elastic segment and A or C denote the glassy ones (Figure 4a, AI-generated). However, diblock structures such as AB, along with BAB triblock copolymers, do not exhibit any reversible elongation, unless the chemical nature of the chains allows for inter- and intra-molecular interactions including ionic interactions [13,14,15], hydrogen bonding [16,17,18,19,20,21,22,23,24,25,26], metal/ligand coordination [27,28,29,30,31,32,33], or π–π stacking [34,35,36,37,38,39,40].

The TPE behaviour of ABA triblock copolymers is based on the thermodynamic incompatibility between the A and B blocks, which leads to microphase separation into a soft matrix and hard microdomains acting as crosslinks. As reported in Section 3.1, the morphology of such a structure is strictly correlated with the ratio between the hard and soft blocks. It is worth noting that the A blocks can belong to the same hard microdomain if the polymer chain forms a loop; conversely, they participate in two different microdomains if the chain acts as a bridge [41]. The latter configuration significantly enhances the mechanical performance of TPEs; therefore, the chemical design of ABA copolymers should favour the formation of bridges [41,42,43]. Indeed, ABC triblock copolymers, containing two different hard blocks, A and C, have been designed to maximise the formation of such bridges over loops (Figure 4b, AI-generated) [44,45,46,47,48,49,50,51,52,53]. Furthermore, due to the thermodynamic incompatibility between the soft and hard segments, as well as between the two different end blocks, the ABC structure leads to various new morphologies [54,55,56].

Another TPE architecture is represented by multiblock copolymers (AB)_n_, which further improve resistance to deformation and cracking without significantly altering phase behaviour [57]. For example, ABABA pentablock copolymers exhibit a lamellar morphology similar to that of AB diblock and ABA triblock copolymers with comparable compositions; however, their distinct mechanical properties arise from the formation of ‘interlocked chain loops’ involving the central A blocks of different chains [58]. For this reason, multiblock architectures typically yield thermoplastic materials; however, it can be highly effective for segmented multiblock TPEs, such as TPUs, COPEs, and COPAs.

While the morphology of linear TPEs is strongly dependent on chemical composition, this effect is less pronounced in branched polymers. Typical branched architectures for TPEs include star, graft, and hyperbranched copolymers. Star copolymers consist of block copolymer chains, namely arms, radiating from a central core. When these arms feature different compositions or molecular weights, the architecture is referred to as a mikto-arm star polymer [59,60]. Graft copolymers consist of a linear polymer backbone with polymeric side chains [61,62]. Their properties are influenced not only by the chemical nature of the polymer chains but also by the molecular weights of both the main and side chains, as well as the distance between the grafted chains [63]. Finally, hyperbranched copolymers feature a high branching density and randomly distributed branches [64]. Recently, other advanced architectures such as dumbbell [65] or pom-pom [66,67,68,69,70,71] copolymers have been used to obtain materials with TPE properties (Figure 4a).

## 3. ABA Block Copolymers as TPEs

### 3.1. General Features

**Figure 5:**
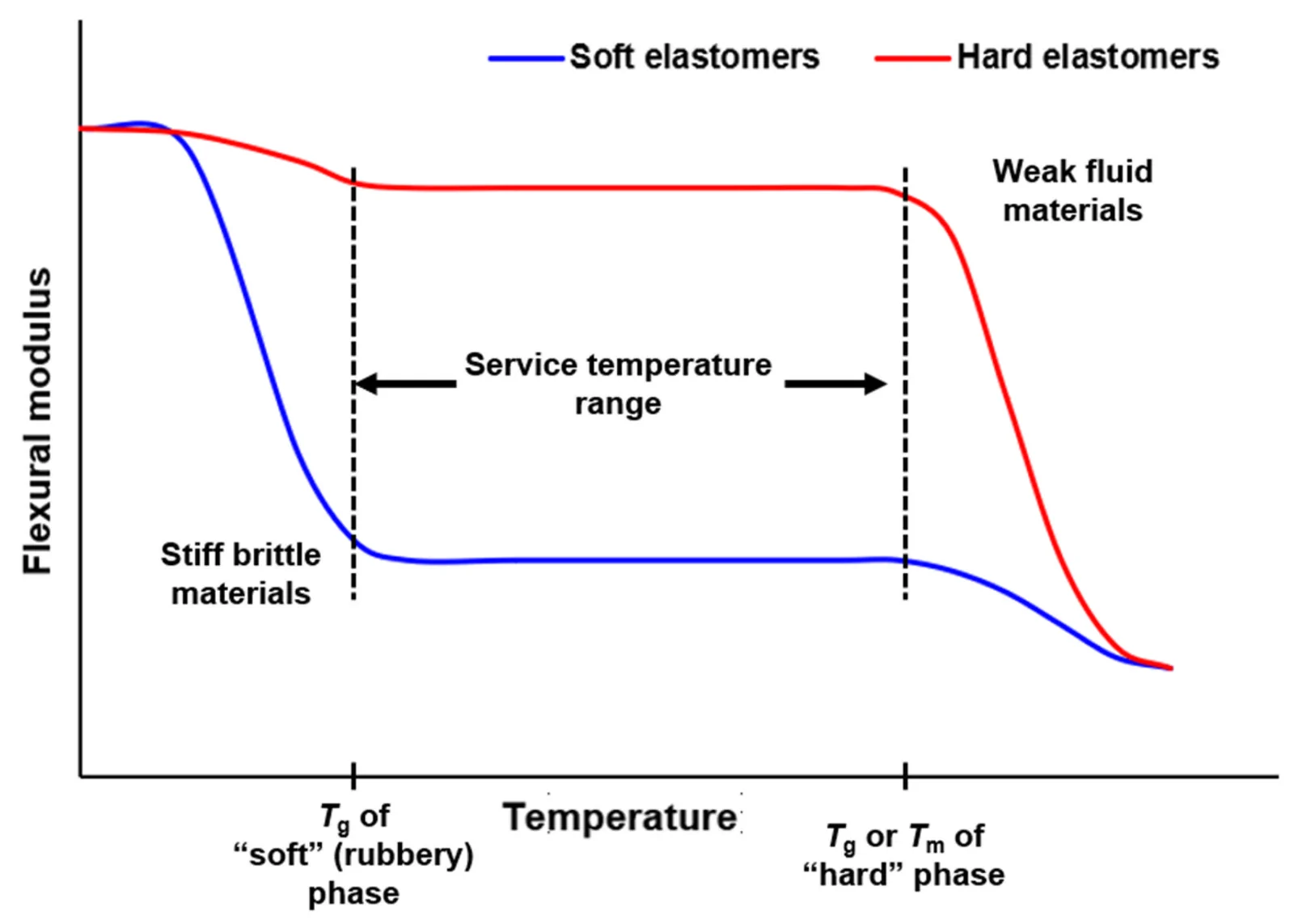
Flexural modulus change with temperature of two generic TPEs.

The ABA block copolymer structure possesses many characteristics of the individual polymer blocks. For example, the glass transition temperature (*T*_g_) is retained by each phase. The external blocks A, with the higher *T*_g_ or melting temperature (*T*_m_), form the so-called hard phase, and above this temperature, the material is a viscous fluid that can be processed. The service temperature range of a TPE, also called rubbery plateau, corresponds to the range between the *T*_g_s of the two blocks A and B. In this range, the hard phase is below its *T*_g_ or *T*_m_ and forms a separate phase organised in microdomains acting as physical crosslinks, being necessary to confer the elastomeric properties to the material. Below the *T*_g_ of the elastomeric component, all the blocks are hard, and thus, the material is stiff and brittle. The overall behaviour is represented by the change in the modulus against temperature, as reported in Figure 5.

It is evident that to achieve final TPE properties, the two components should neither be mutually soluble nor form a homogeneous phase; however, it has been observed that they should also be neither completely incompatible. In fact, TPEs are made of polymer blocks that are compatible in the way that there is a slight degree of mixing or interfacial bonding. Generally, materials are considered miscible when the Gibbs free energy of mixing, expressed as in Equation (1), is negative:

(1) $$\Delta G_{m}=\Delta H_{m}-T{\Delta S}_{m}$$

where $\Delta H_{m}$ and ${\Delta S}_{m}$ are the enthalpy and entropy of mixing, respectively.

**Figure 6:**
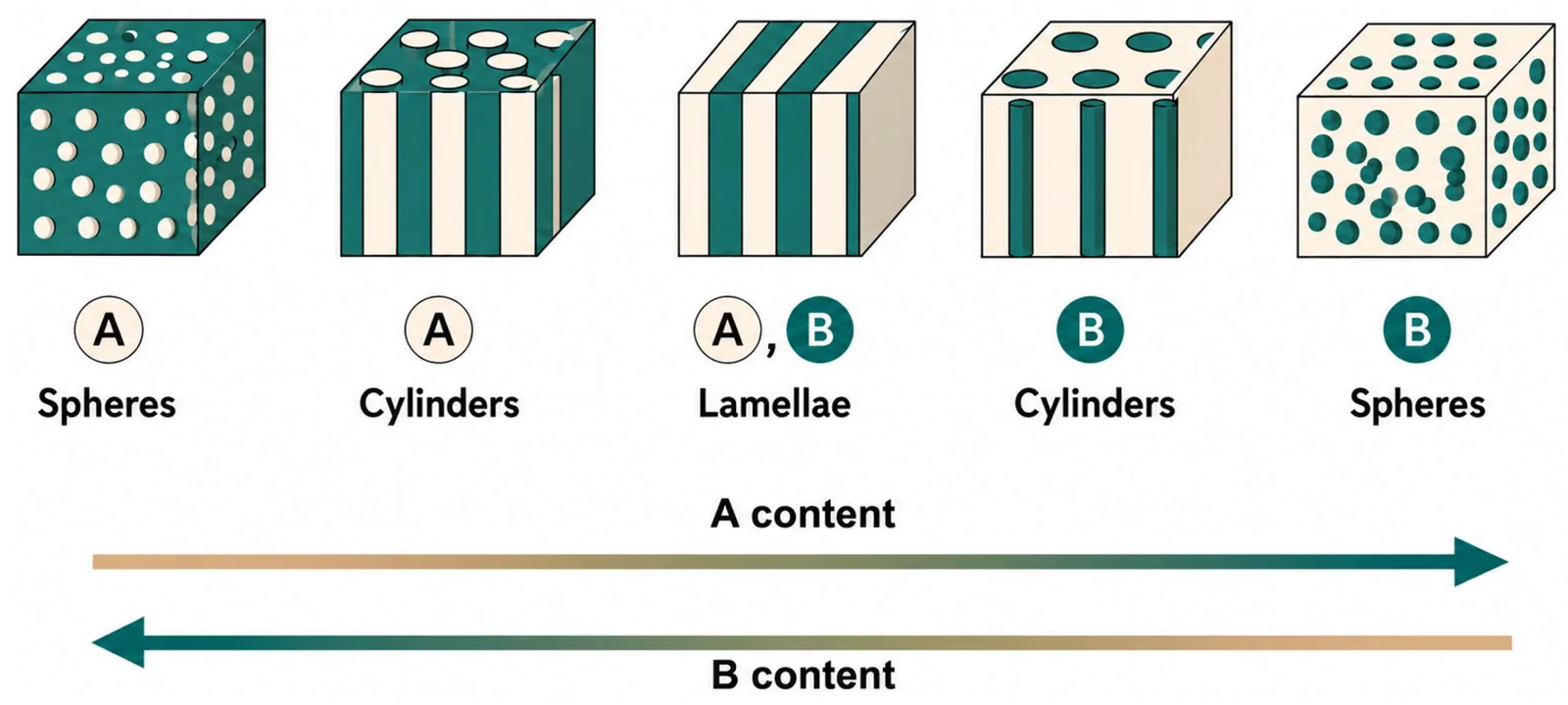
Morphology changes of ABA copolymer block as a function of composition. (The conceptual image was adapted from Ref. [3] and generated using OpenAI’s GPT-5.6 Luna model. The authors have reviewed and edited the output and take full responsibility for the content of this publication).

Another approach to evaluate the polymer mixing thermodynamics is given by the Flory–Huggins model, originally developed for polymer solutions but can be extended to polymer–polymer mixtures. For a system of two polymers, the Flory–Huggins free energy density is [72,73]:

(2) $$f\left(\varphi \right)=\;\frac{\varphi }{N_{A}}\;\ln\varphi +\;\frac{1-\varphi \;}{N_{B}}\;\ln\left(1-\varphi \right)+\chi \varphi (1-\varphi )$$

where *φ* is the volume fraction of one component, and *N_A_* and *N_B_* are the number of monomer units of polymers A or B. The interaction parameter χ represents an effective thermodynamic parameter reflecting the energetic compatibility between polymer components and generally depends on temperature and composition. In its simplest interpretation, χ is related to the enthalpic contribution to mixing (ΔH_m_) and arises from the energetic difference between unlike and like intermolecular contacts, which are primarily associated with van der Waals interactions. Consequently, phase separation is promoted by increasing χ, indicating poorer energetic compatibility, and by increasing the molecular weight (N), which reduces the entropy of mixing [74,75]. The separation of the domains in an ABA structure is not visible, and the material appears transparent [76]; nonetheless, optical transparency is also dependent on the crystallinity, size of the domain, refractive index contrast, and processing conditions. Morphological investigation by electron microscopy and other techniques has shown that the organisation of the phase separation depends on the relative quantity of the two polymers. Low amounts of hard copolymer will be dispersed in elastomeric domains in the shape of spheres and, for higher contents, progressively distributing as cylinders and lamellae, with the latter detectable when approximately equal volume fractions of A and B are present (Figure 6, AI-generated) [42,43]. It should be noted that the phase separation greatly impacts the mechanical properties of the material; hence, not all morphologies are desirable in the case of TPEs (vide infra).

### 3.2. ABA Block Copolymers Synthesis

**Scheme 1:**
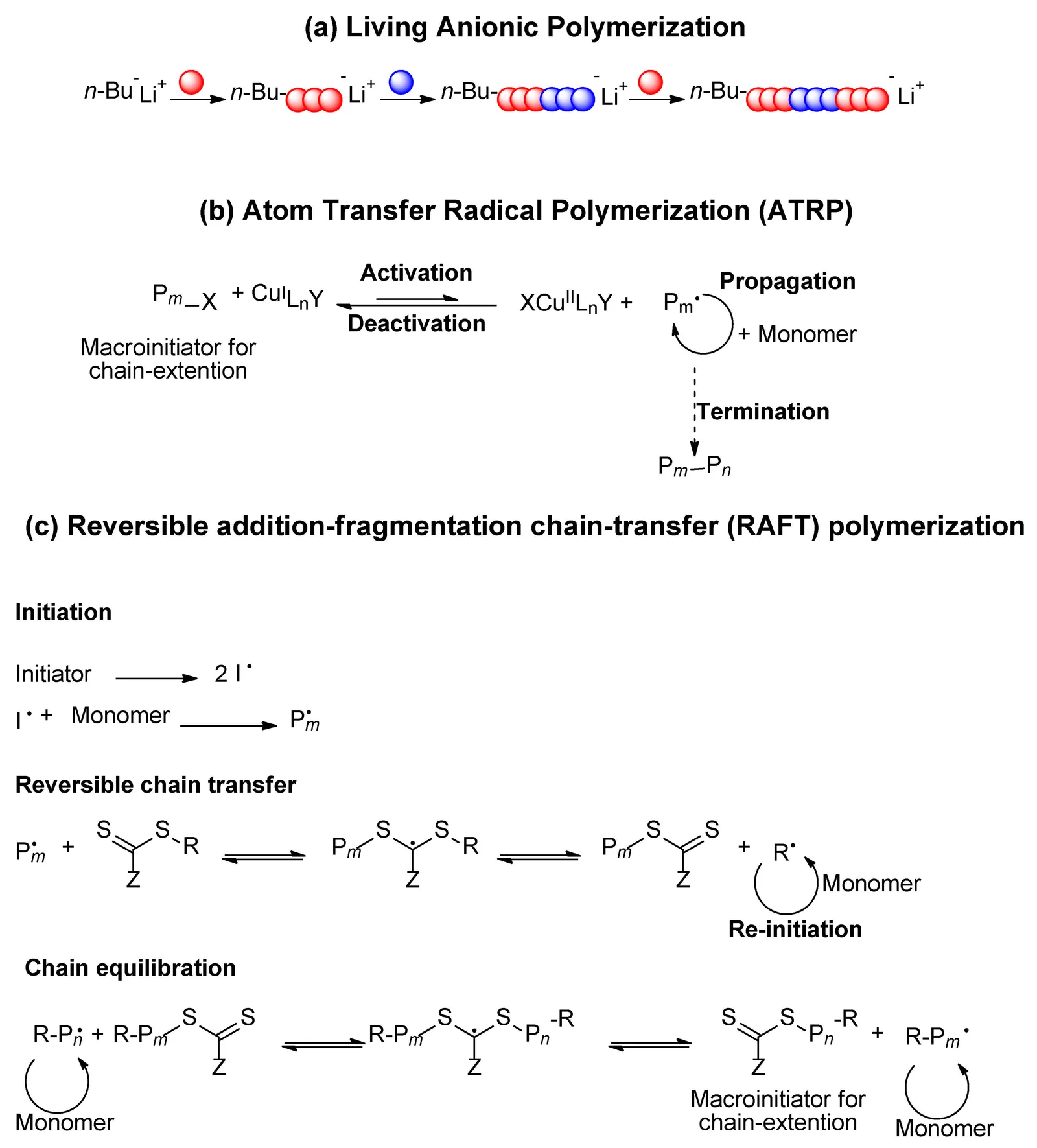
Mechanisms of the main living polymerisation technique towards ABA copolymers [77,78,79,80].

Copolymers having ABA structures are generally synthesised by means of living polymerisation techniques that, depending on the nature of the propagating species, can be classified in ionic (anionic and cationic) and radical living polymerisation (Scheme 1). Anionic living polymerisation requires strong nucleophilic initiators (i.e., *n*-butyl lithium) that, by attaching the first monomer unit, generates a stable carbanion able to propagate. In the absence of impurities (adventitious water and oxygen), no chain termination occurs, and block copolymers can be produced by sequential addition of different monomers (Scheme 1a) [61]. Living cationic approaches proved more complex than the anionic counterpart, in terms both of experimental conditions and scope of monomers [77].

Among radical polymerisation methods, both free- and controlled radical polymerisation (CRP) techniques can be employed; in this review, we will refer mainly to the latter and, in particular, to Atom-Transfer Radical Polymerisation (ATRP) and Radical Addition–Fragmentation Chain-Transfer (RAFT) Polymerisation. ATRP is based on a reversible equilibrium between active radical species and dormant species. It involves an initiator (generally an alkyl halide), a transition metal complex with a ligand, and monomers. Halogen transfer generates radicals that propagate the polymer chain, while the equilibrium ensures control over the process (Scheme 1b) [78]. RAFT uses a radical initiator, monomer, and a chain transfer agent (CTA) and proceeds through a degenerative chain transfer mechanism. The CTA, defined by its R and Z groups, governs polymerisation control and can introduce specific functionalities. Different types of CTAs (e.g., trithiocarbonates and dithioesters) are used, and by modifying R and/or Z groups, polymers with tailored end functionalities (α, ω, or telechelic) can be produced (Scheme 1c) [79,80]. Both CRP processes can be employed in the preparation of ABA block copolymers by progressive chain extension of previously isolated macroinitiators (i.e., central blocks having active termini on either side of the chain).

### 3.3. Characterisation Techniques

To fully characterise and understand the thermoplastic elastomer behaviour, a combination of techniques that elucidate both their thermal and mechanical properties, as well as their microstructural organisation are used. Concerning the structure, essential analyses include ^1^H and ^13^C NMR spectroscopies and Size Exclusion Chromatography (SEC). While NMR spectroscopy provides information on chemical composition, SEC allows for the determination of molecular weight and molecular weight distributions, parameters that influence phase separation, chain entanglement, and consequently, mechanical and rheological properties of the material. Indeed, the molecular weight of the individual blocks affects the size and stability of the microphase-separated morphology and the effectiveness of the hard domains as physical crosslinks. It has to be noted that although routine NMR experiments can also provide, to some extent, information on the polymer molecular architecture, advanced techniques (i.e., DOSY [81]) are often required to unambiguously characterise the material. Thermogravimetric Analysis (TGA) can assess the thermal stability and composition of polymers by monitoring weight changes as a function of temperature. The weight losses observed can be attributed to decomposition, oxidation, or volatilisation of components. The polymer thermal stability is used to define the maximum service temperature at which materials can be applied retaining its mechanical properties. Differential scanning calorimetry (DSC) is crucial for identifying *T*_g_s of the hard and soft segments, as well as any melting or crystallisation transitions. For example, in the case of semicrystalline polymers, *T*_m_ and *T*_c_ define the temperature at which the crystallites melt in heating scans or crystallise in cooling scans, respectively. *T*_g_ represents, instead, a key parameter of an amorphous polymer and is related to the change from glassy to melt or vice versa. This phenomenon is detected as a step in the heat flow trace since there is a heat capacity increase for the melt. For block copolymers, the appearance of distinct *T*_g_ values indicates phase separation behaviour; instead, a single, broadened *T*_g_, would suggest miscibility between the blocks. For semicrystalline blocks, the enthalpy of melting or crystallisation peaks can quantify the crystalline content of the hard segments, which directly impacts the material’s mechanical strength. Moreover, as described before, the temperature range between the lower and upper *T*_g_ values defines the range in which TPEs express their unique properties due to physical crosslinks (ca. −50 °C up to 180 °C, depending on the type of material). In some cases, DSC proves limited sensibility, and the determination of the *T*_g_ values requires the use of rheological methods such as Dynamic Mechanical Analysis (DMA), which can sometimes exhibit higher sensitivity over DSC by measuring the temperature- and frequency-dependent viscoelastic response, in particular changes in the storage modulus (*E′*), loss modulus (*E″*), and damping factor (tan δ), providing insights into molecular mobility, chain dynamics, and network stability. A major limitation of this technique resides in the amount of material necessary to prepare free-standing specimens for the analysis; indeed, such quantities may not always be readily available on a laboratory scale. Nevertheless, to overcome this issue, alternative specimen configurations have been developed [82].

Information on characteristic domain spacings, correlation length, and structural organisation in phase-separated block copolymers can also be obtained by small-angle X-ray scattering (SAXS). It can reveal how block composition, molecular weight, segregation strength, and processing conditions affect the size and organisation of hard domains, which act as reversible physical crosslinks. On the other hand, reduced spatial resolution, a lack of local information, and the requirement of a certain extent of prior knowledge of the sample’s morphology are common drawbacks associated with this technique. Atomic force microscopy (AFM) is a high-resolution imaging technique that allows for the examination of polymer surfaces at the nanoscale. In AFM, a sharp tip mounted on a cantilever scans the surface of the polymer, and the deflection of the cantilever is measured to create a topographical map. The analysis can reveal surface morphology, roughness, and nanostructures, providing insights into the polymer’s mechanical properties and interactions with other materials [83]. Finally, tensile testing is fundamental for evaluating the mechanical performance of TPEs. Stress–strain curves provide information about the material’s tensile strength, elongation at break, and modulus, which are directly related to the interplay between the hard and soft segments. Hysteresis loops can also be obtained and analysed to understand the energy dissipation or recovery behaviour of the elastomer.

Overall, the combination of all such techniques provides a comprehensive approach to understanding TPEs. Correlating molecular architecture and thermal transitions with nanoscale morphology, viscoelastic behaviour, and mechanical response clarifies how the organisation and physical crosslinking of hard domains control stiffness, strength, elasticity, hysteresis, energy dissipation, and recovery. This approach offers a comprehensive understanding of polymer characteristics, enabling researchers to tailor polymers for specific applications and improve their performance in various industries.

## 4. Fossil-Based ABA Block Copolymers

### 4.1. Styrenic Block Copolymers (SBCs)

**Figure 7:**
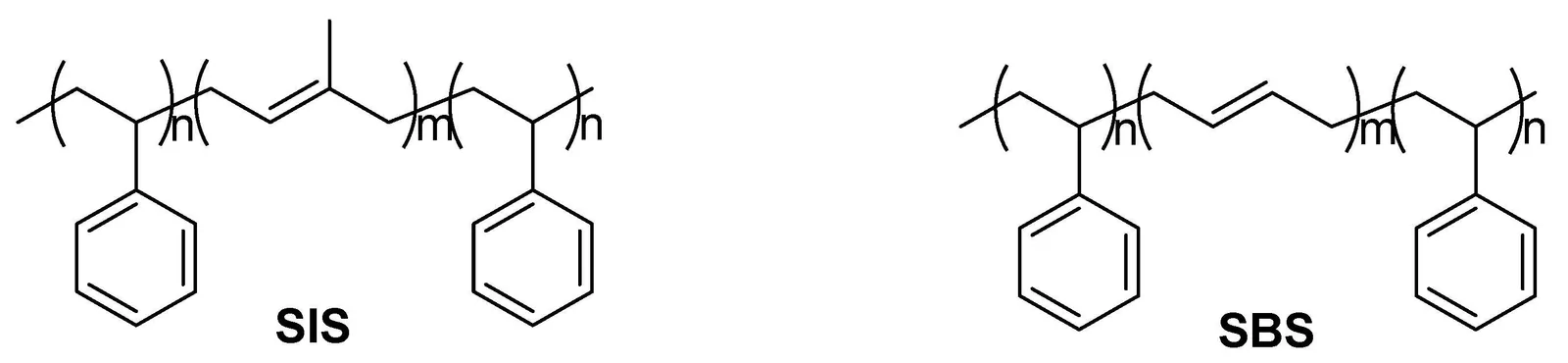
Structure of poly(styrene-block-isoprene-block-styrene) (SIS) and of poly(styrene-block-butadiene-block-styrene) (SBS).

Styrenic block copolymers (SBCs) are TPEs based on an ABA triblock structure in which the hard phase A is often composed by polystyrene (PS) (*T*_g_ ~ 100 °C) while the elastomeric domain B is made by a polydiene, like polybutadiene (PB) (*T*_g_ ~ −90 °C) or polyisoprene (PI) (*T*_g_ ~ −56 °C) [84]. These block copolymers are generally synthesised by anionic polymerisation, requiring harsh experimental conditions such as the use of high-vacuum procedures and high-purity starting reagents to prevent accidental termination caused by the presence of impurities (Section 3.2). Nevertheless, two among the most diffused TPEs, namely poly(styrene-*block*-butadiene-*block*-styrene) (SBS) and poly(styrene-*block*-isoprene-*block*-styrene) (SIS) (Figure 7), are currently produced on a commercial scale by anionic polymerisation of the common monomers styrene, butadiene, and isoprene.

**Figure 8:**
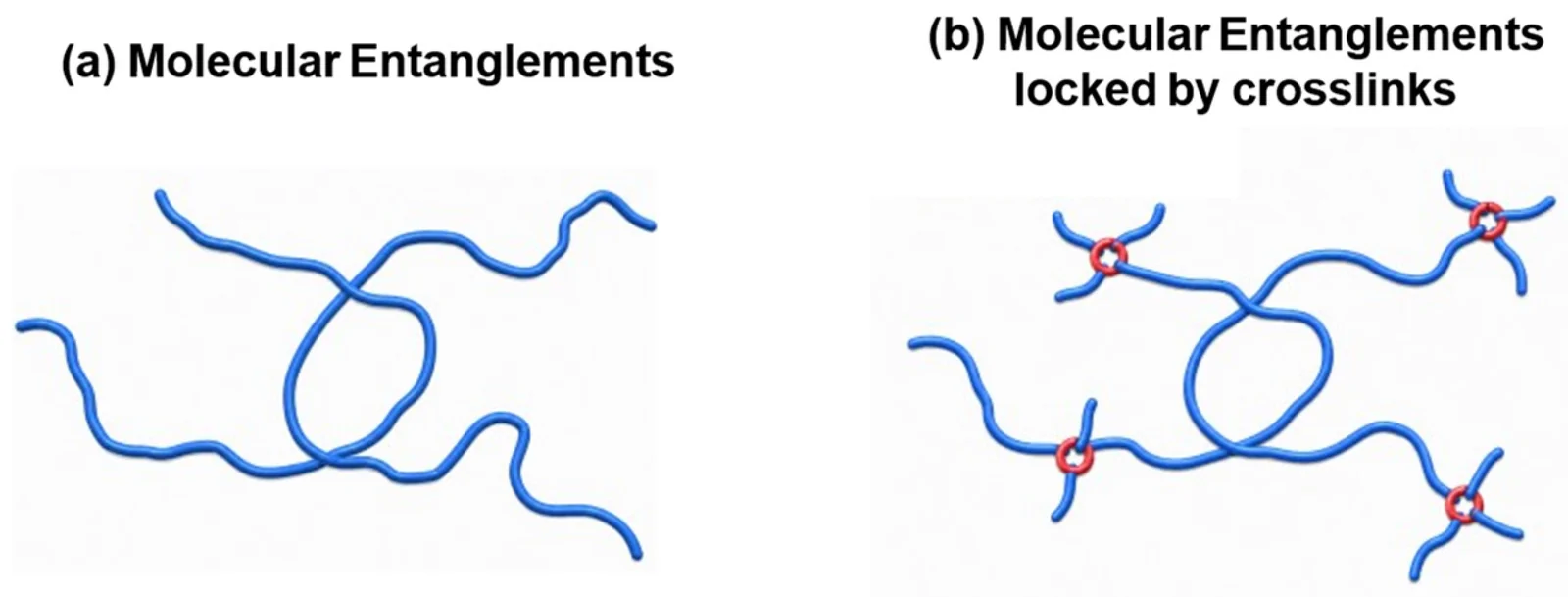
Representative molecular entanglements in a high *M*_W_ polymer (**a**) and the same locked by crosslinks (**b**). (The conceptual image was adapted from Ref. [8] and generated using OpenAI’s GPT-5.6 Luna model. The authors have reviewed and edited the output and take full responsibility for the content of this publication).

It is worth noting that while the sequence SIS confers to the material the typical thermoplastic and elastomeric properties, structures like ISI or IS are not able to create a continues network and an efficient phase separation, and as a consequence, they do not produce TPEs [3]. As mentioned in Section 3.1, the phase ratio of the hard–soft components has an impact on the morphology and thus also on the final properties. Indeed, in the case of SBCs, the spherical morphology is preferred to gain good mechanical properties. As the polystyrene content increases, the material became stiffer and harder up to a glassy thermoplastic behaviour. The tensile moduli and tensile strength values, at a constant content of polystyrene, yet enough to have phase separation, do not depend on the molecular weight of the polymers; however, the elastic modulus highly depends on the nature and lengths of the middle block. An important parameter is considered to be the molecular weight between entanglements, *M*_e_, defined as the average molecular weight of a chain segment between intermolecular junction points. A larger *M*_e_ indicates fewer entanglements per polymer chain, while a smaller *M*_e_ means more entanglements. For elastomeric materials, more entanglements mean higher resistance to deformation and improved elongation at break. In fact, the entanglements act like temporary crosslinks, allowing the material to recover its shape when the stress is removed (Figure 8, AI-generated). Generally, commercial SBCs have molecular weight well above the *M*_e_ of the middle block (*M*_e_ for polybutadiene and polyisoprene is 1.7 and 6.1 kg/mol, respectively).

Further developments of the classic TPEs such SBS and SIS are the derivative hydrogenated polymers, like poly(styrene-*b*-ethylene-butylene-*b*-styrene) SEBS and poly(styrene-*b*-ethylene-propylene-*b*-styrene) SEPS, that overcome the weakness due to the unsaturated double bond of the middle block. In fact, polyisoprene of SIS and polybutadiene of SBS can undergo oxidation and UV degradation that reduce the lifetime and stability of the materials. Concomitantly, the hydrogenation can induce crystallinity, which can affect some mechanical properties. Another approach involves the use of saturated polyisobutylene (IB) as the middle block. The final triblock copolymer SIBS is synthesised in one pot by living cationic polymerisation. More advances in the field have recently been reviewed by Maji and Naskar [85].

### 4.2. Acrylic-Based TPEs

**Figure 9:**
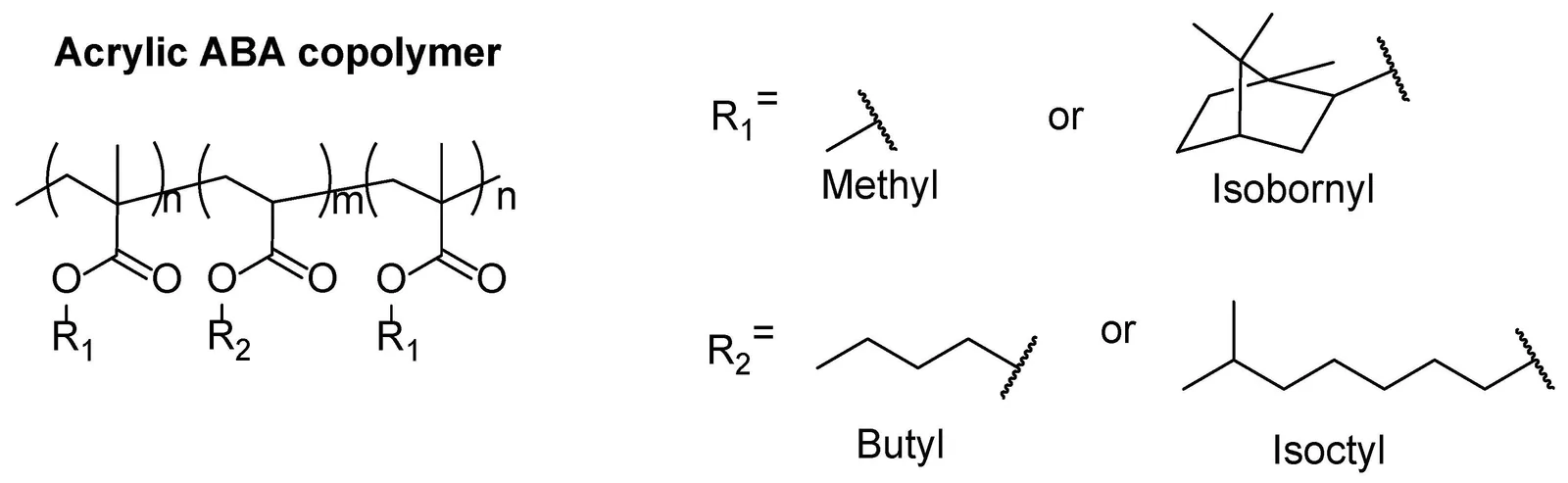
General structure of the full acrylic ABA copolymers.

A promising alternative to SBCs overcoming the stability issues is represented by the fully acrylic-based block copolymers. These copolymers usually have the same ABA structure of the SBCs, in which A and B serve as the hard and the soft phase, respectively. For example, commercial products are available with the ABA structure in which poly(n-butyl acrylate) (PnBA) (*T*_g_ ~ −50 °C) acts as the soft domain while poly(methyl methacrylate) (PMMA) (*T*_g_ ~ 100 °C) is the hard domain (Figure 9) [86].

Acrylate living anionic polymerisation results are challenging due to the side reaction involved in the process that does not guarantee good polymerisation control. Better control can be achieved by using bulky pendant groups that prevent the side reactions. Furthermore, processes such as alcoholysis allowed Tong, et al. to synthetise P(MMA-*b*-nBA-*b*-MMA) from P(MMA-*b*-tBA-*b*-MMA) [87]. The full acrylic TPE P(MMA-*b*-nBA-*b*-MMA) is a commercial available product that is sold by Arkema with the tradename Nanostrength^®^ and is produced using nitroxide-mediated polymerisation (NMP) [88]; instead, Kuraray Co. Ltd. developed an anionic polymerisation system that use a Lewis base and di-phenoxyalkyl aluminium [89]. Both these polymerisation systems allow for sequential monomer addition without affecting the living character of the process. Although triblock copolymers can be made with the same composition by different synthetic procedures, the mechanical performances of the resulting materials can also be different. For instance, Tong et al. compared P(MMA-*b*-nBA-*b*-MMA) copolymers made by living anionic polymerisation and by ATRP. Even though molecular characteristics of the two copolymers were essentially the same, they observed low storage modulus and poor ultimate tensile properties for the material prepared by ATRP. The only relevant difference was found to be a slightly broader polydispersity of the PMMA outer blocks when using ATRP. This parameter was considered as responsible for the lower performance due to the formation of a less effective network [90].

Fully acrylic TPEs can have advantages over SBCs in terms of optical transparency, oil resistance, adhesion, weatherability, low viscosity, printability, compatibility with fillers, and abrasion resistance [86,91]. Moreover, an extensive range of structurally different copolymers can be produced due to the variety of acrylate and methacrylate monomers with different pendant groups. For example, poly(isobornyl methacrylate) (*T*_g_ ~ 200 °C) was used as the glassy segment and poly(2-ethylhexyl acrylate) (*T*_g_ ~ −65 °C) [92] or poly(isoctyl acrylate) (*T*_g_ ~ −50 °C) [93] as the soft segment. However, achieving comparable mechanical properties to those of SBCs is still challenging, mainly due to the high molecular weights between entanglements of polyacrylates as middle blocks (Table 1).

**Figure 10:**
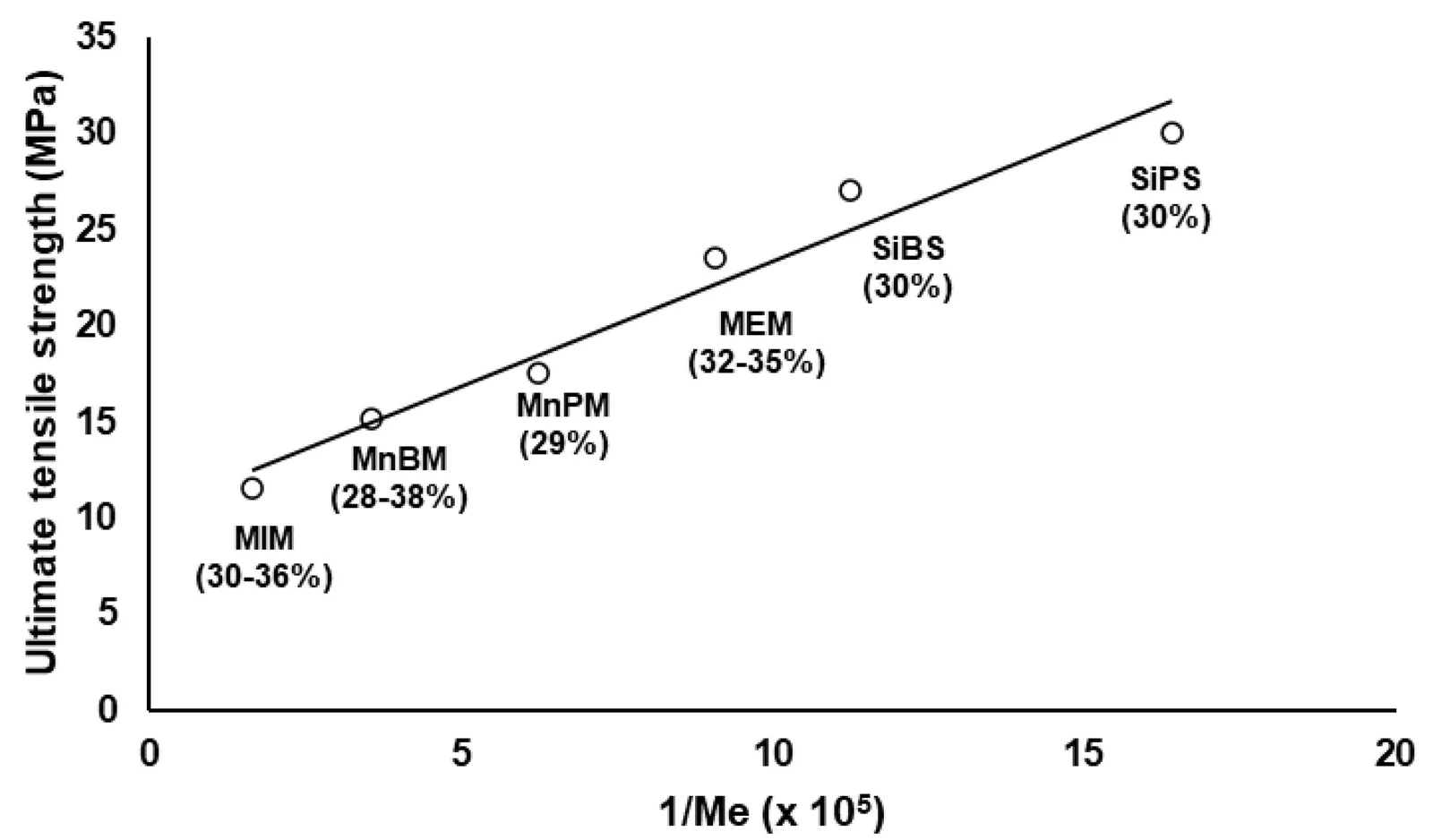
Ultimate tensile strength versus 1/*M*_e_. [M = PMMA; I = poly (iso-octyl acrylate); nB = poly(n-butyl acrylate); nP = poly(n-propyl acrylate); E = poly(ethyl acrylate); S = PS; iB = poly(iso-butylene); iP = polyisoprene)] [adapted from Ref. [94]].

Comparing TPEs with similar structure, the general observation is that with polybutadiene as the middle block and, as external blocks, poly(styrene) (SBS) or poly(methyl methacrylate) (MBM), elongation can easily reach 1000%. Upon using poly(n-butyl acrylate) PnBA as the soft block, the elongation of the ABA triblock is generally lower than that observed in the presence of poly(butadiene), either using, as A blocks, styrene (for P(S-*b*-nBA-*b*-S), where elongation is less than 600% [95]), or PMMA (for P(MMA-*b*-nBA-*b*-MMA), where elongation is 500% [94]). Moreover, a decrease in the tensile strength was also observed when going from the classic SBCs to the acrylate ABA TPEs, as shown by the extensive investigation undertaken by Tong and Jérôme (Figure 10) [96].

To overcome these weaknesses, different strategies have been investigated. One of these employs the use of a multi-graft structure. Lu and coworkers reported the synthesis of a PnBA-graft-PMMA thermoplastic elastomer by using living anionic polymerisation to obtain a PMMA macromonomer and RAFT polymerisation for the synthesis of PnBA. This approach allowed for elongations up to 1700% [97]. In fact, like star copolymers, a graft architecture can help to have more entanglements between the hard segments, promote more crosslinks, and create superior mechanical strength [98]. Further advancement of polymerisation techniques and of supramolecular chemistry have inspired new approaches to have materials with superior properties: the introduction of dynamic covalent bonds allowed for the formation of self-healing or shape memory materials [99]. Supramolecular chemistry has been applied to PS-*b*-PnBA diblock copolymers to create an ABA triblock copolymer by dimerisation of an end-functionalised external block with 2-ureido-4-pyrimidinone. The final material exhibits the mechanical properties of a thermoplastic elastomer and the dynamic self-healing capabilities of supramolecular materials [100]. Notably, PMMA-PnBA copolymers have favourable interchain van der Waals forces, which can also lead to self-healing behaviour [101,102].

## 5. Bio-Based TPEs

In the last decades, significant efforts have been devoted to the development of sustainable and renewable materials, aiming at reducing the dependence on non-renewable resources and at minimising the overall environmental impact [103]. Indeed, much research has also been focusing on the development of bio-based TPEs. These materials are derived from renewable biomass sources and can offer a more sustainable alternative to the traditional ones.

As previously discussed, TPEs can be classified into different types, and for nearly all categories, numerous examples have been reported of efforts for the transition to bio-based alternatives. Notably, studies on bio-based TPEs consider thermoplastic elastomers with structures based on polyurethane [104], polyamide [105,106], or polyester [107,108], typically obtained through polycondensation reactions. These bio-based TPEs show properties comparable to their petroleum-based counterparts.

**Scheme 2:**
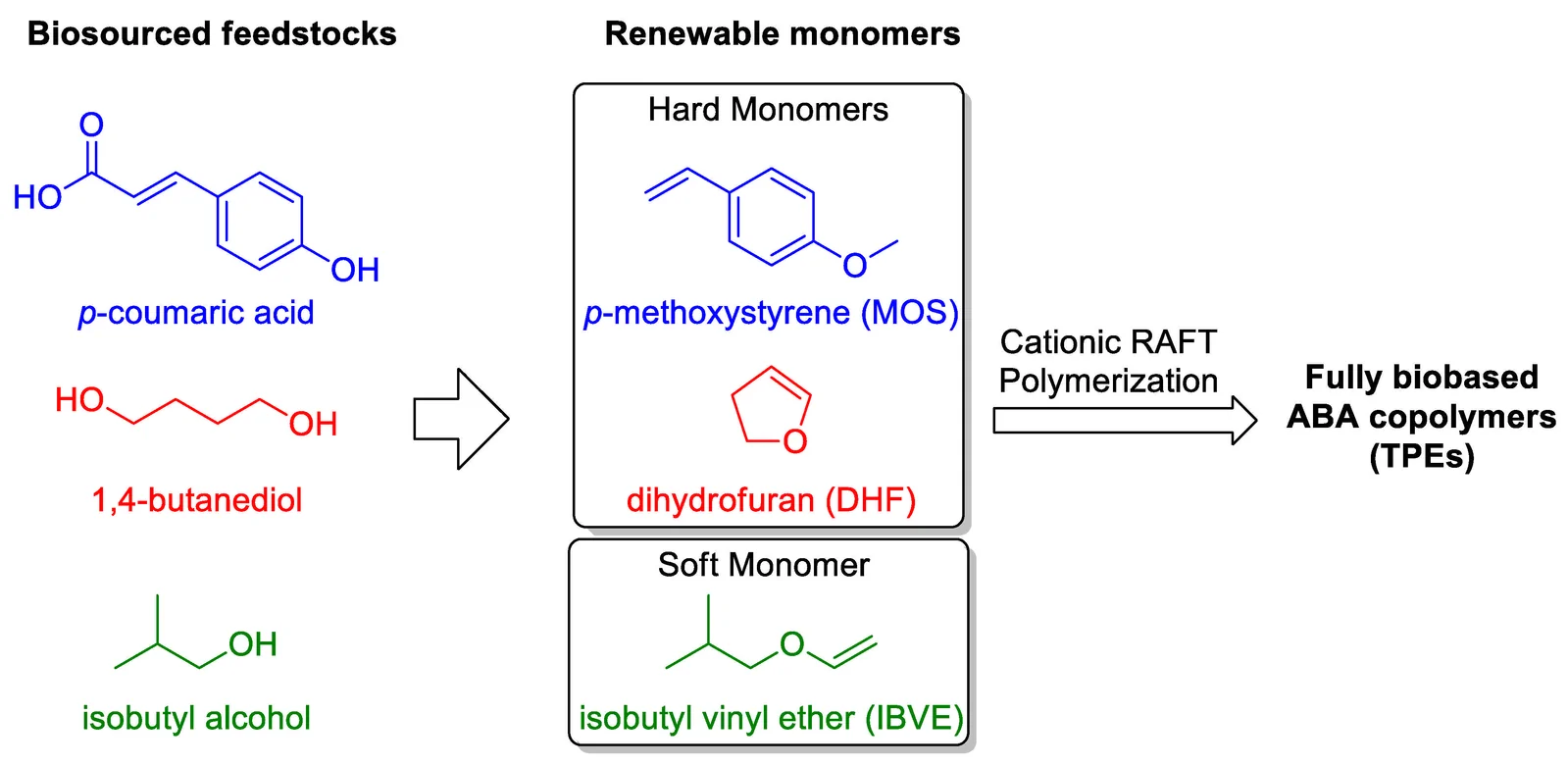
Synthesis of fully bio-based ABA block copolymers exhibiting TPE behaviour [110].

To date, ABA structures remain the most common among bio-based TPEs, likely due to the well-understood phase separation mechanism and established controlled polymerisation techniques that facilitate relatively straightforward and precise synthesis. In building an ABA-structured TPE, one hard and one soft polymer are essential. Researchers are therefore exploring biomass-derived monomers with these properties. Renewable sources such as plant sugars, vegetable oils, lignin, and terpenes provide a range of suitable building blocks. Moreover, the sustainability of the final material depends on several factors, including the avoidance of biomass that competes with food or feed production, as well as the development of nontoxic and sustainable synthetic processes for both monomers and polymers. Additionally, new approaches and materials are worth exploring to expand knowledge in this field and create new material combinations with improved performance [109]. An elegant example of fully bio-based ABA copolymers exhibiting TPE properties was recently reported by Fors and coworkers [110]; in this scenario, *p*-methoxystyrene (MOS) obtained from renewable *p*-coumaric acid and dihydrofuran (DHF) derived from biosourced 1,4-butanediol were identified as the hard monomers, while bio-based isobutyl vinyl ether (IBVE) was the soft block precursor (Scheme 2). The ABA structures were obtained by RAFT polymerisation using a difunctional CTA and ferrocenium tetrafluoroborate (FcBF_4_) as a catalytic chemical oxidant. Without isolation of the middle block, telechelic PIBVE was then chain extended with either MOS or DHF to yield P(MOS-*b*-IBVE-*b*-MOS) or P(DHF-*b*-IBVE-*b*-DHF), respectively. The *T*_g_ of the soft block was found to be −10 °C, while that of the hard segment was 105 °C and 126 °C for PMOS and PDHF, respectively. Mechanical tests revealed that while both copolymers performed as TPEs, PDHF-based materials exhibited superior strength, elongation, and recovery than their *p*-methoxystyrene analogues.

### 5.1. Terpene-Based TPEs

**Figure 11:**
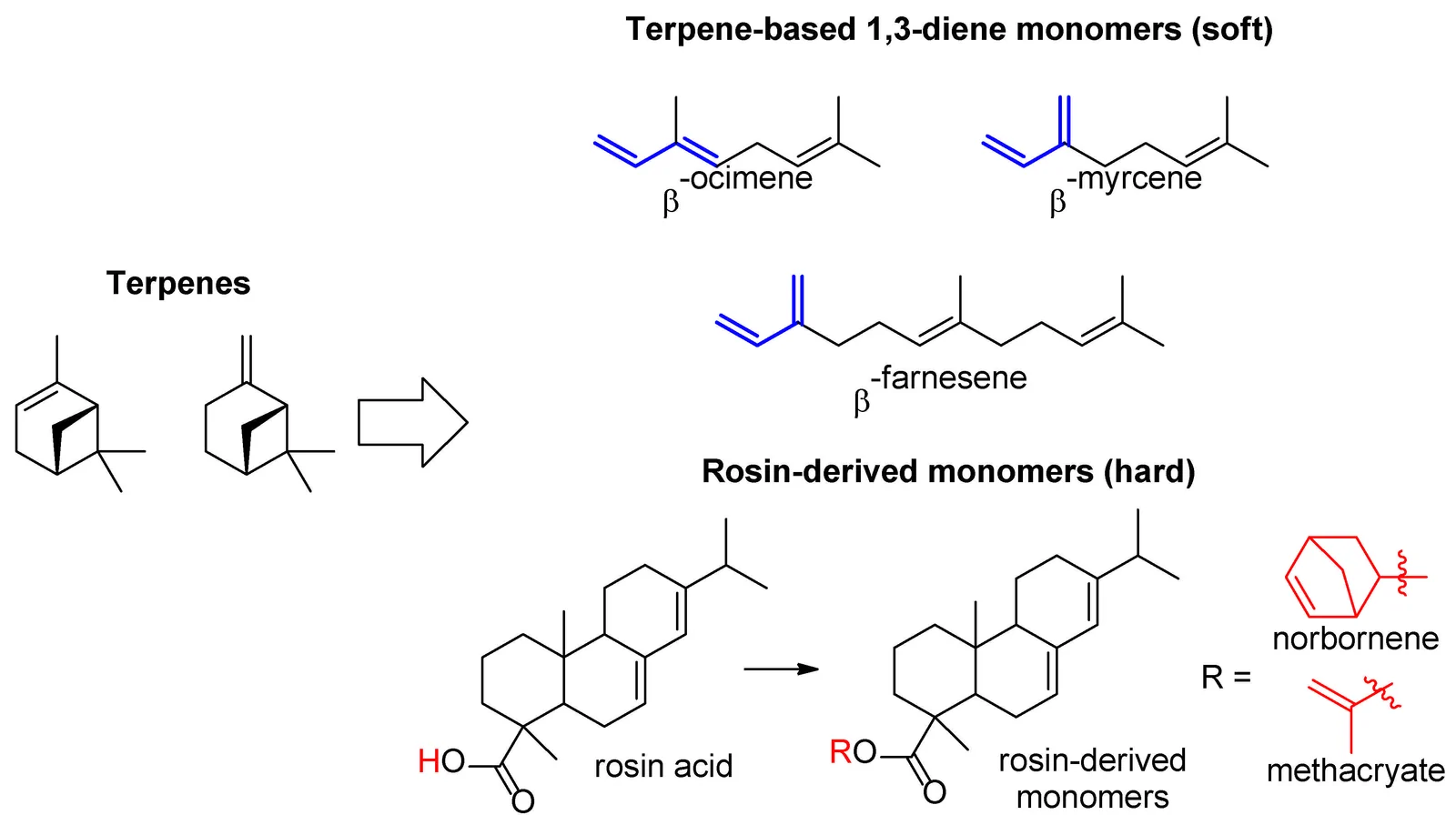
Examples of hard and soft terpene-derived monomers [109,111,112,113,114].

Terpenes are natural organic compounds produced mainly by plants; although structurally diverse, many terpenes contain unsaturated carbon–carbon bonds, which can be exploited in polymerisation. For example, β-pinene, extracted from turpentine oil, can be converted into several polymerisable monomers containing a 1,3-diene moiety, hence resembling the structure of isoprene, a ubiquitous monomer for soft blocks (vide supra). Indeed, terpenes such as β-ocimene, β-myrcene (Myr), or β-farnesene (Far) have recently emerged as bio-derived alternatives to fossil-based dienic monomers (Figure 11) [111,112]. Moreover, hard monomers can be obtained from esterification of terpene-derived rosin acid upon introduction of a polymerisable group such as methacrylate or norbornene [113,114].

Terpene-based TPEs synthesised from β-myrcene and a β-pinene derivative (nopadiene) [115], via living polymerisation and with a tapered structure, resulted in a copolymer with thermal and mechanical properties comparable to standard SBCs, including glass transition temperatures of −45 °C and 100 °C, elongation at break (ε_b_) of 1200%, and tensile strength (σ_b_) of 2.5 MPa [116]. A fully terpene-based ABA triblock copolymer was synthesised by sequential anionic polymerisation of α-methyl-p-methylstyrene (AMMS) and β-myrcene, in which the former and the latter served as the hard and the soft monomer, respectively. Interestingly, the thermal properties of the renewable TPS proved better than those exhibited by the conventional, fossil-based SBS or SIS counterparts due to its higher service temperature (up to 160 °C) [117].

**Figure 12:**
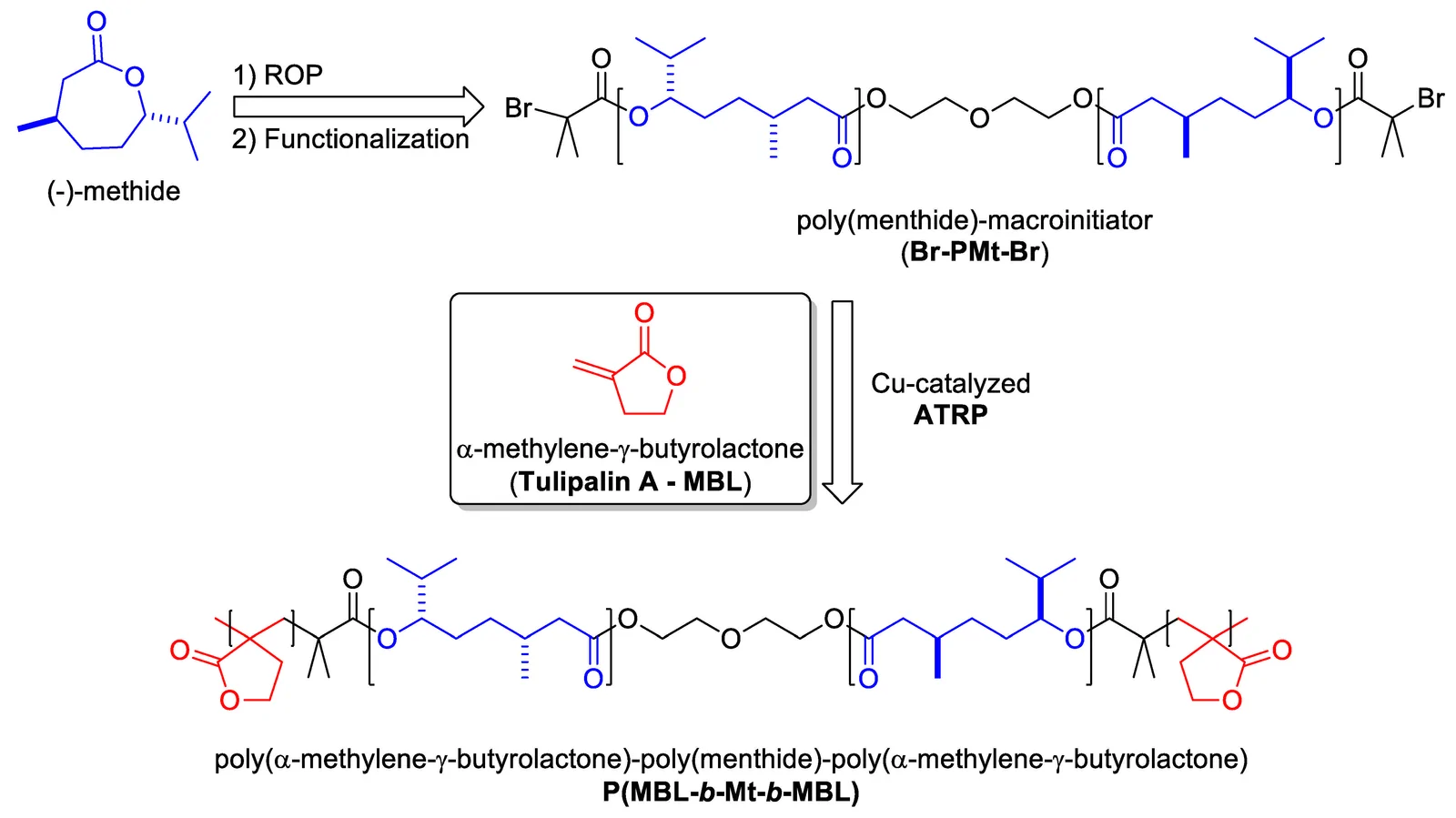
Menthide-Tulipalin A-based TPE.

The anionic copolymerisation of β-farnesene and styrene was recently disclosed by Frey and coworkers [118]. Due to strongly different reactivity of the two monomers, tapered (block-like), rather than random, copolymers were attained. Tri- and pentablock copolymers with low dispersity were synthesised in one pot; moreover, such materials exhibited phase-separated nanostructures similar to those of polyisoprene-based copolymers (e.g., lamellar and cylindrical), although the bulkier side chain of poly(β-farnesene) determined some differences in chain configurations, as well as a reduction in chain entanglement, leading to poorer mechanical properties (i.e., toughness). Nevertheless, the lower solution viscosity of the β-farnesene-based copolymers allowed for improved processability. Another example of ABA triblock uses menthide (Mt), a menthol derivative, as the soft block obtained by Ring-Opening Polymerisation (ROP). The hard block was then formed by ATRP of Tulipalin A (MBL), a γ-butyrolactone derivative (Figure 12) [119]. The resulting triblock copolymer, P(MBL-*b*-Mt-*b*-MBL), demonstrates good thermal and mechanical properties, with elongation up to 1300% and tensile strength close to 13 MPa. By contrast, when butyl acrylate (nBA) is used as a soft block, the triblock P(MBL-*b*-nBA-*b*-MBL) showed significantly lower performance (ε_b_ = 100, σ_b_ = 0.6) [120].

**Figure 13:**
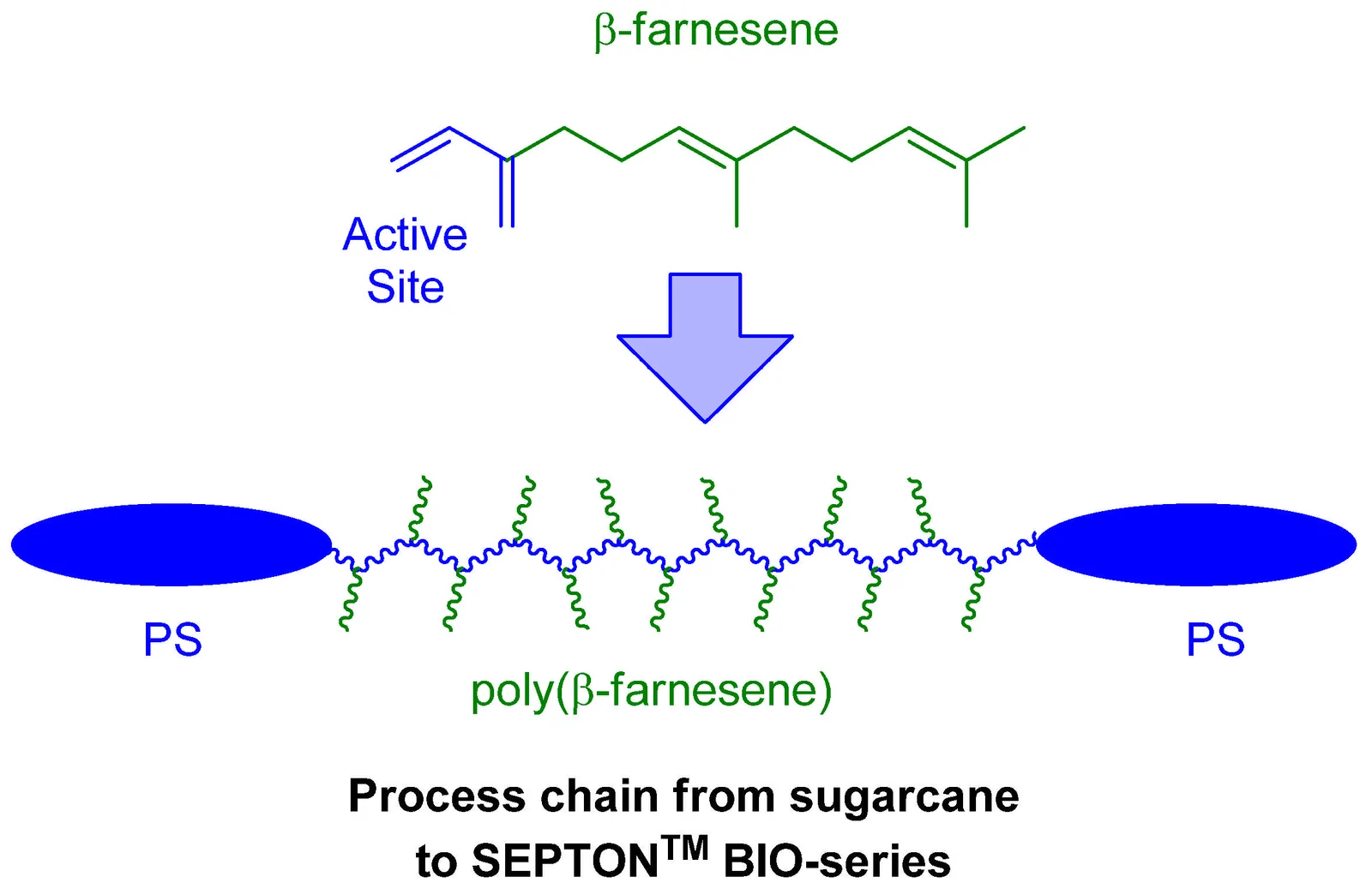
Structure of SEPTON^TM^ BIO.

Terpene-based TPEs also find commercial applications. To this end, SEPTON^TM^ BIO (Kuraray company), having the hydrogenated structure of styrene-*b*-β-farnesene-*b*-styrene, exhibits mechanical properties comparable to or even superior to that of oil-based hydrogenated SBCs (Figure 13) [121].

### 5.2. Bio-Based Acrylic TPEs

In the field of ABA triblock copolymers with acrylic structures made with at least one monomer derived from biomass, many examples have been reported in the literature [122]. The rising interest in these materials is driven by their advantages over other TPEs, along with the variety of available monomers and from the tolerance of controlled radical polymerisation techniques towards such bio-derived building blocks. Acrylic bio-based materials generally have low TPE mechanical performance, compared to SBCs, yet exhibit recognised characteristics as pressure-sensitive adhesives, as blend compatibilisers, or as toughening agents.

**Figure 14:**
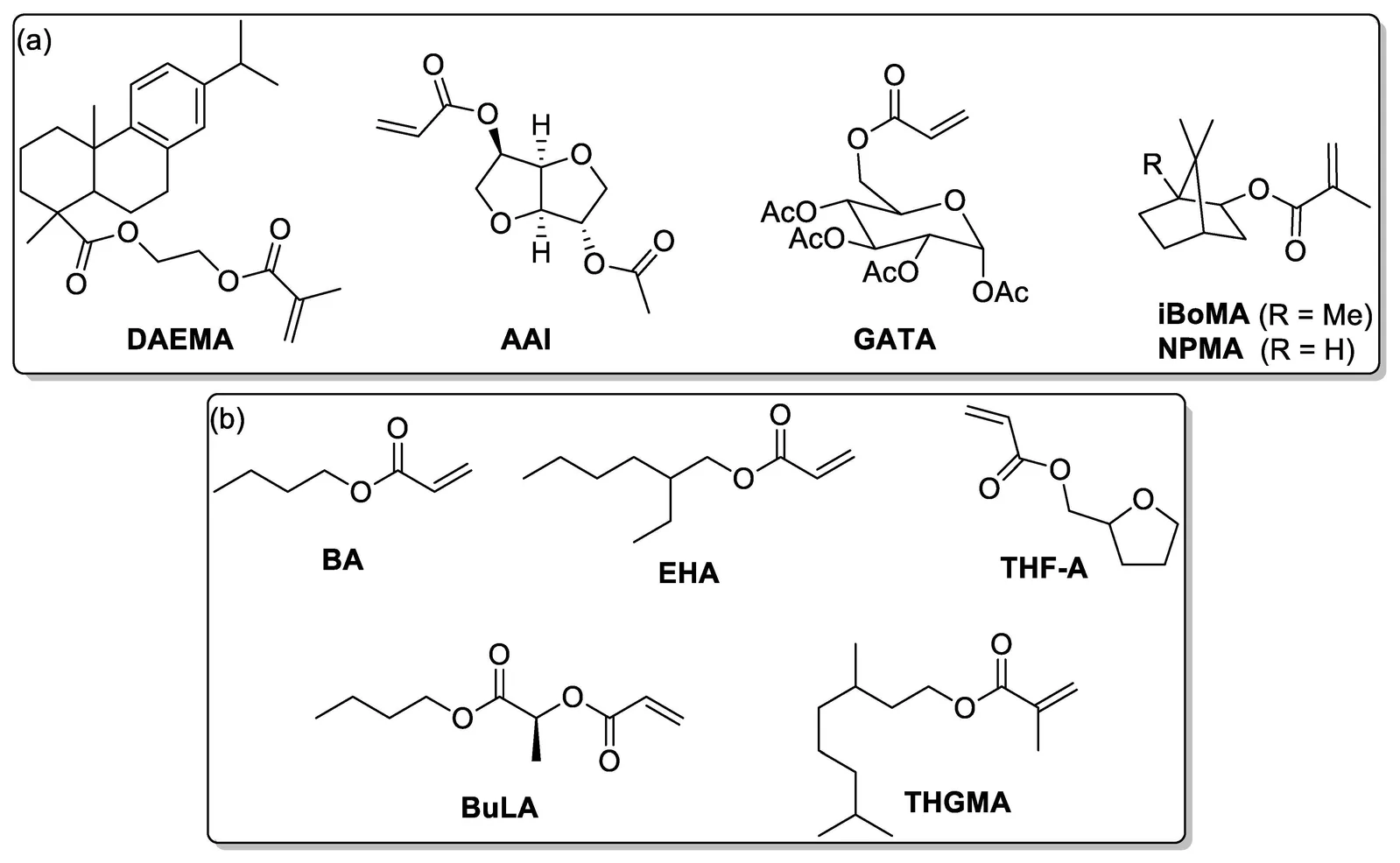
Structure of terpene-derived (meth)acrylates used as hard (**a**) and soft (**b**) monomers.

As observed before, n-butyl acrylate (nBA) is a widely used monomer for the soft part of acrylic oil-based TPEs. Several authors have used PnBA as a soft oil-based block [86,95,120] to build and study ABA triblock copolymers with different hard terpene-based domains (Figure 14). When a rosin derivative, the dehydroabietic ethyl methacrylate (DAEMA), was used as a hard monomer, the triblock P(DAEMA-*b*-nBA-*b*-DAEMA) showed well-defined microphase-separated morphology and relatively good TPE properties such as elongation at break (ε_b_) of 240% and tensile strength at break (σ_b_) of 1.5 MPa (*T*_g_s −43 and 95 °C) [113]. The sugar derivatives acetylated acrylic isosorbide (AAI) or glucose-6-acrylate-1,2,3,4-tetraacetate (GATA) used as external parts, in an ABA structure synthesised via RAFT, allow for the formation of a P(AAI-*b*-nBA-*b*-AAI) or P(GATA-*b*-nBA-*b*-GATA) triblock with a good service temperature (*T*_g_s −45 °C and >100 °C). Mechanical properties improved when a soft block with higher molecular weight (*M*_n_) was used; an elongation of about ε_b_ = 600% instead of ε_b_ = 200% was reached by using a middle block with *M*_n_ = 110 kg/mol instead of 56 kg/mol; higher *M*_n_ was also responsible for an increase of the tensile strength (σ_b_ from 0.6 to 0.8 MPa for GATA and from 0.06 to 6.5 MPa for AAI) [123,124].

Using AAI as a hard block but combined with a soft block different from nBA, specifically 2-ethylhexyl acrylate (EHA), the P(AAI-*b*-EHA-*b*-AAI) triblock copolymer evidenced worse TPE properties (ε_b_ = 35% and σ_b_ = 0.02) and worse adhesive performance [125]. Moreover, the same hard block, AAI, used in a triblock structure with another soft block, butyl lactate acrylate (buLA), a derivative of lactic acid, allows one to obtain P(AAI-*b*-buLA-*b*-AAI) copolymer. This TPE exhibited competitive adhesive tape properties and decent mechanical characteristic (*T*_g_s −20 and 100 °C, σ_b_ = 0.3 MPa, and ε_b_ = 250%). Note the high different performance of these mentioned copolymers sharing AAI as the same hard block and with the different soft blocks nBA (ε_b_ = 600%), buLA (ε_b_ = 250%), or EHA,(ε_b_ = 35%). The lowest performance and, in particular, lowest elongation of the EHA copolymer was attributed to the higher average molecular weight between entanglements of PEHA. Properties vary considerably depending on the chosen soft block, making the choice of the soft monomer critical to final performance.

Other terpene-derived hard monomers have been explored, including, very recently, a series of ABA triblock copolymers having, along with MMA, the bio-based isobornyl methacrylate (iBoMA) as the hard monomer and nBA and bio-based tetrahydrofurfuryl acrylate (THF-A) as the soft block precursors [126]. The synthesis of the hard blocks showed excellent control, yielding polymers with narrow dispersities. PMMA exhibited a *T*_g_ around 100 °C, whereas PiBoMA displayed significantly higher *T*_g_s in the range of 190–200 °C. By copolymerising MMA and iBoMA at different feed ratios, the thermal properties could be finely tuned, affording copolymers with *T_g_*s between 130 and 155 °C. The bio-derived THF-A soft block exhibited a low *T*_g_ of approximately −11 °C, confirming its suitability for elastomeric applications. Well-defined high-molecular-weight ABA architectures, including P(MMA-*b*-nBA-*b*-MMA), P(iBoMA-*b*-nBA-*b*-iBoMA), and P(iBoMA-co-MMA)-*b*-(nBA)-*b*-P(iBoMA-*co*-MMA), were prepared with good control. However, the isolation of the fully bio-based triblock terpolymers proved more challenging, particularly due to difficulties associated with the short soft block length. The resulting materials retained thermoplastic elastomer behaviour after five recycling cycles at 180 °C for 30 min while maintaining an essentially unchanged storage modulus (G′) in the MPa range and demonstrating preservation of the triblock architecture. Furthermore, rheological and scattering analyses confirmed microphase separation into distinct hard and soft domains, with the PiBoMA-based triblock systems notably exhibiting an accessible order–disorder transition. Similarly, RAFT polymerisation was employed for the preparation of ABA copolymers consisting of β-pinene-derived poly(nopinyl methacrylate) (PNPMA) as the external hard blocks and poly(tetrahydrogeraniol methacrylate) (THGMA) as the central soft block [127]. The hard monomers, nopinyl methacrylate and nopinyl acrylate, exhibit high glass transition temperatures, with *T*_g_ values of approximately 160 °C for PNPMA and 74 °C for PNPA, while the soft poly(tetrahydrogeraniol methacrylate) block possesses a low *T*_g_ of −13 °C, as determined by DMA analysis. SAXS and AFM analyses confirmed clear phase separation within the materials, consistent with well-defined block copolymer morphologies. Preliminary mechanical and adhesive testing demonstrated promising material performance, with probe tack experiments showing acceptable adhesive behaviour over repeated cycles alongside encouraging strength characteristics. Tetrahydrogeraniol methacrylate has also been used as the soft monomer in ABA TPEs featuring styrene as the hard block scaffold [128].

**Figure 15:**
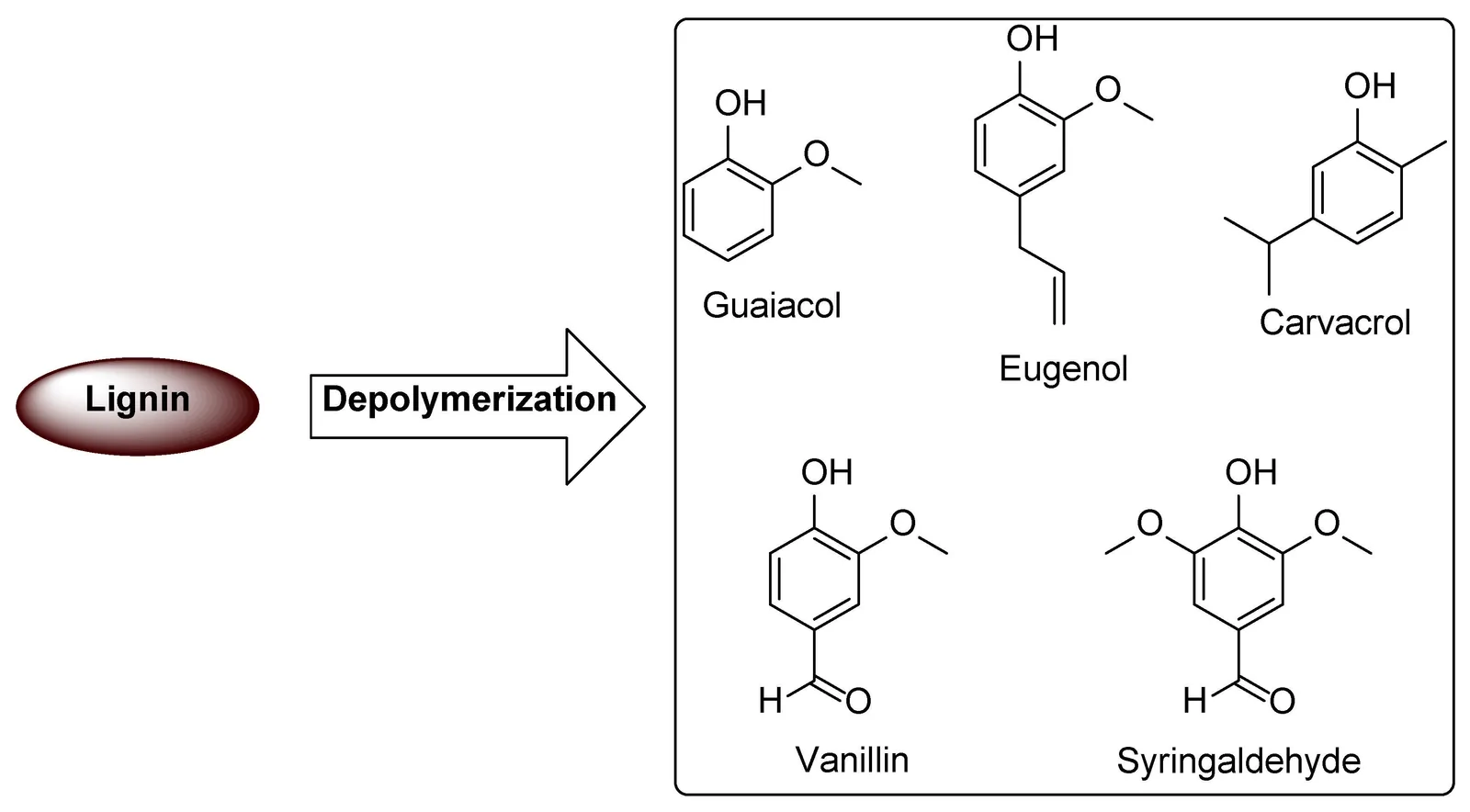
Phenolic building blocks obtainable from lignin depolymerisation.

Phenols derived from the depolymerisation of lignin, namely guaiacol, eugenol, carvacrol, vanillin, and syringaldehyde, have recently emerged as promising building blocks for hard monomers (Figure 15) [129,130,131,132,133].

**Figure 16:**
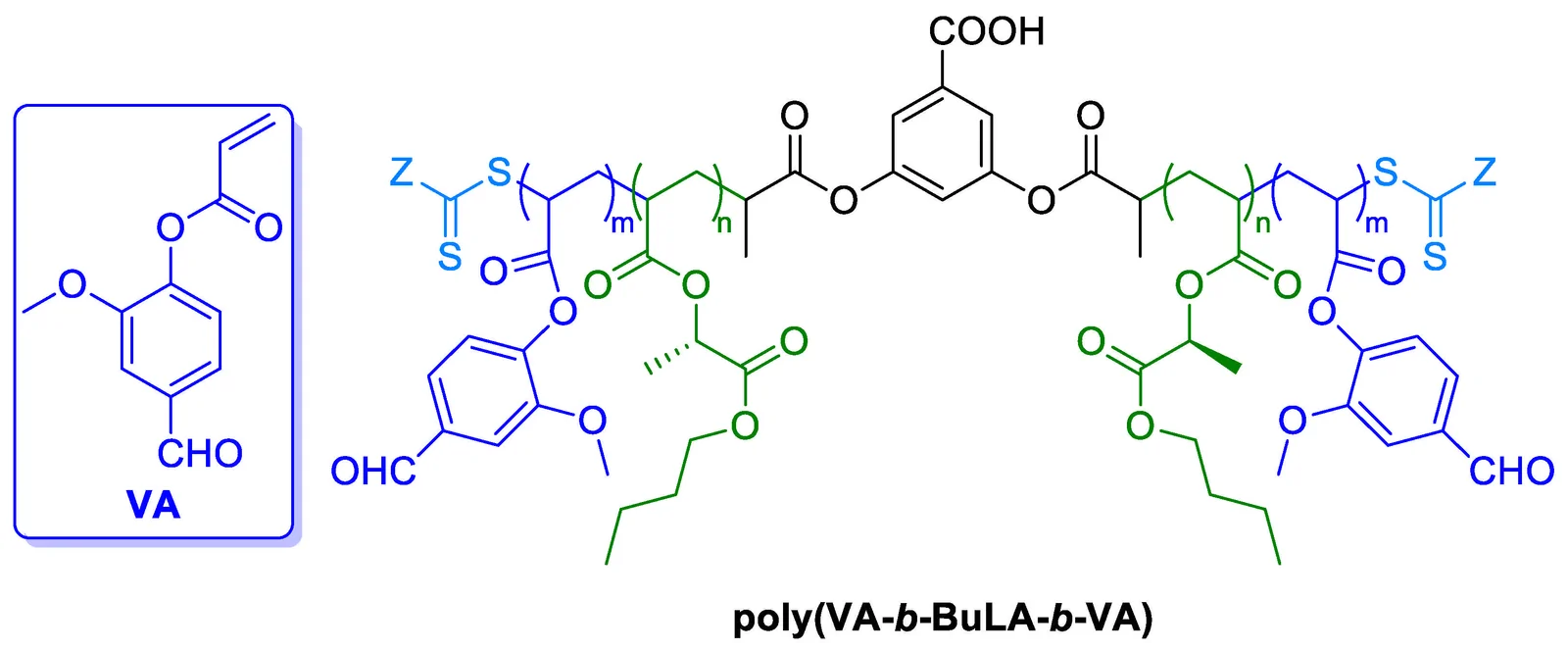
ABA block copolymers with a lactic-acid-based middle block acrylic monomer and VA as the external block [134].

With respect to TPEs, vanillin acrylate (VA) has been employed in combination with buLA in the synthesis of P(VA-*b*-buLA-*b*-VA), affording comparable elongation (ε_b_ = 250%) and slightly improved tensile strength (σ = 0.8 MPa) than the previously discussed P(AAI-*b*-buLA-*b*-AAI) copolymers [134] (Figure 16).

Furthermore, Epps’ group reported the use of 4-propylsyringol and 4-propylguaiacol (meth)acrylate, with n-butyl acrylate as the soft domain. The block copolymers exhibited excellent thermal and adhesion properties, suitable for application as a pressure-sensitive adhesive (*T*_g_s −45 and *n.d*., σ_b_ < 1 MPa, ε_b_ ca. 500%) [135]. They also used vanillin methacrylate combined with lauryl methacrylate (LMA), a fatty acid-based derivative, to study the diblock structure, attaining tuneable flow behaviour and good thermal properties [136]. Similarly, poly(guaiacyl acrylate) (PGA) was employed as the glassy end block in linear ABA triblock copolymers, in which poly(methyl acrylate) (PMA) served as the soft middle block [137]. Remarkably, even a very low PGA end-block content (<5 mol%) led to substantial improvements in mechanical performance, increasing the tensile strength by approximately 3–7 times while maintaining high elongation at break. These enhanced properties were attributed to the formation of microphase-separated morphologies, as confirmed by calorimetric and scattering analyses. In addition to improved mechanical behaviour, the triblock elastomers also demonstrated enhanced thermal stability compared to the corresponding parent homopolymers. A further example is about P(GM-*b*-HL-*b*-GM), a *mixed* structure with the soft phase obtained via ROP of hexalactone (HL) and the hard phase from the RAFT polymerisation of guaiacyl methacrylate, yielding a biodegradable copolymer with interesting final properties depending on the soft block length (σ from 1 to 2 MPa and ε from 900 to 3000%) [138].

**Figure 17:**
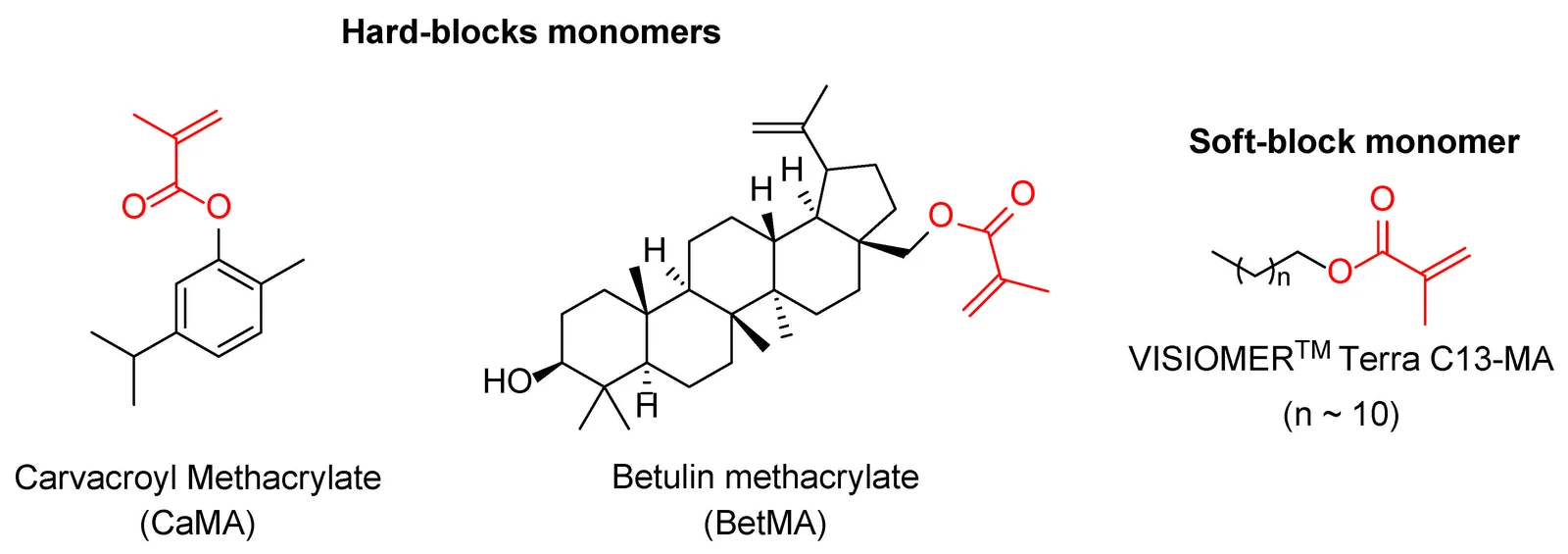
Monomers employed in the RAFT-synthesised bio-based ABA copolymers.

Very recently, some of us developed fully bio-based triblock copolymers via RAFT polymerisation of betulin methacrylate (BetuMA) or carvacroyl methacrylate (CaMA) and VISIOMER^®^ Terra C13 (ET13) (a lauryl methacrylate analogue) as the hard and soft monomers, respectively [139] (Figure 17). Thermal analyses indicated a *T*_g_ of −50 °C for the middle soft block, while the poly(carvacroyl methacrylate) exhibited a *T*_g_ of around 60 °C; on the other hand, no *T*_g_ could be detected for poly(BetuMA). AFM analysis revealed well-defined phase separation, which was further supported by DSC and WAXS measurements, confirming the formation of distinct hard and soft domains essential for elastomeric performance. Tensile testing demonstrated elastomeric behaviour across all block copolymers, with the BetuMA-based BEB materials outperforming the CaMA-based CEC analogues. In particular, BEB copolymers achieved high tensile strength and elongation at break, even at lower hard block contents, highlighting the superior reinforcing capability of BetuMA and the strong influence of hard block chemistry on mechanical performance. Samples with moderate glassy block content exhibited the best balance between strength and extensibility, while increasing hard block content improved stiffness at the expense of elongation.

**Figure 18:**
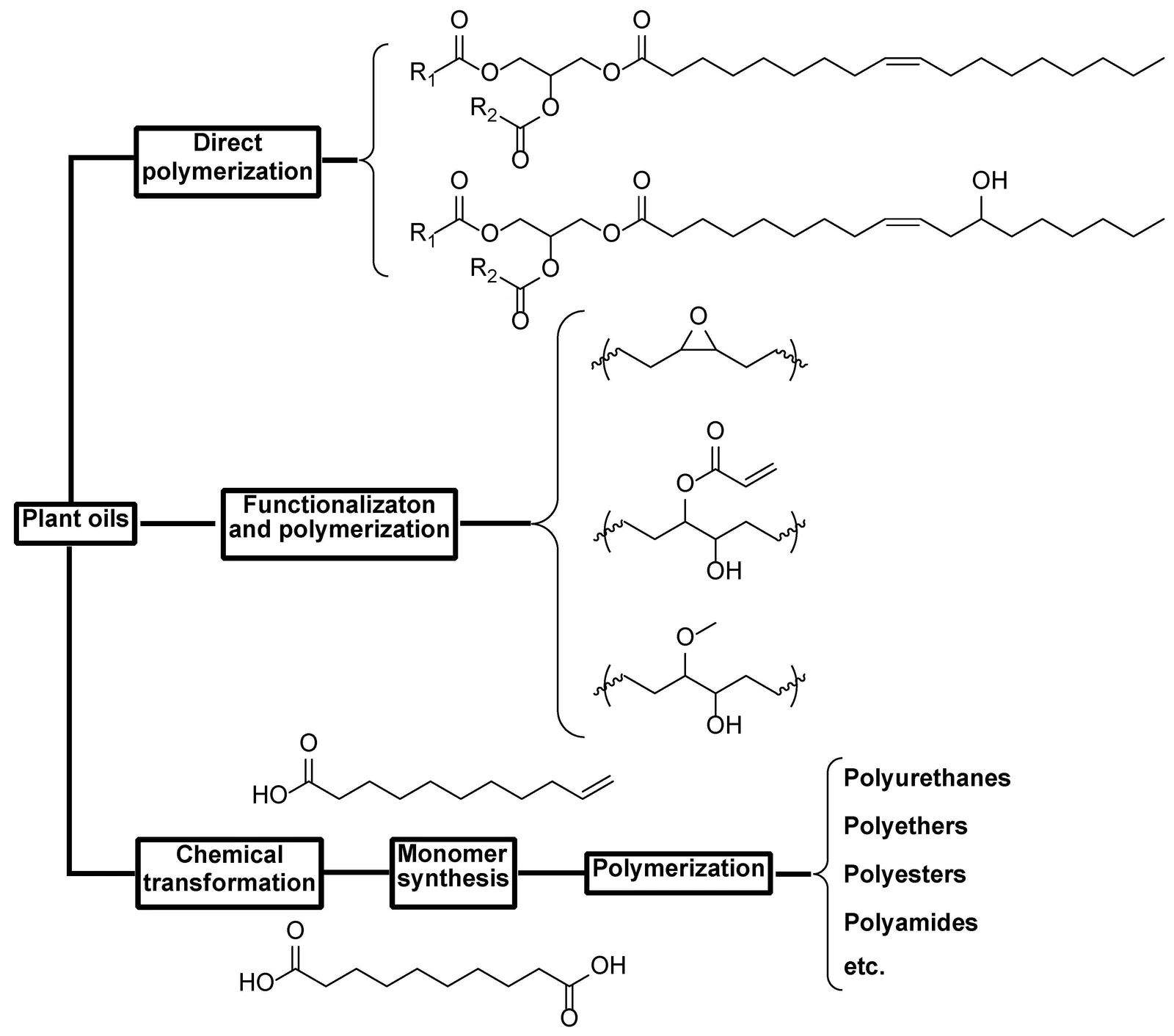
General strategies for the synthesis of polymers from plant oils.

Plant oils and their fatty acids, due to their low toxicity, availability, and relatively low price, are particularly attractive as a building block source (Figure 18) [140,141]. One strategy for the valorisation of plant oils involves the carboxylic acid group of a fatty acid that can be transformed into a hydroxyl group [142] and then converted to a (meth)acrylate group.

For example, lauryl methacrylate (LMA), a derivative of lauryl alcohol, has been reported by different authors as a monomer capable of forming the soft part in the TPE ABA structure [143]. When an oil-based monomer was used for the hard part, like methyl methacrylate (MMA), the final triblock has good thermal service temperature (*T*_g_s −55 and 100 °C) yet rather modest mechanical performance (σ_b_ = 1–3 MPa, ε_b_ ca. 80–100%) [144]. Instead, when the bio-based monomer acetylsalicylic ethyl methacrylate (As) was used to make the P(As-*b*-LMA-*b*-As) triblock, an elongation up to 250% and σ_b_ = 0.44 MPa were achieved [145]. Nevertheless, their low performance compared to SBCs was explained above all because of the high entanglement molecular weight of PLMA (*M*_e_ = 225 kg/mol). Improved mechanical performance is achieved using LMA as the soft domain with, as a hard block, oil-based tertbutyl methacrylate (tB). In fact, the copolymer P(tB-*b*-LMA-*b*-tB) reached an elongation of 350% with a maximum strength of 2.4 MPa, and these improvements were tentatively attributed by authors to a higher *T*_g_ of PtB and a partial mixing of the two polymer phases [144].

**Figure 19:**
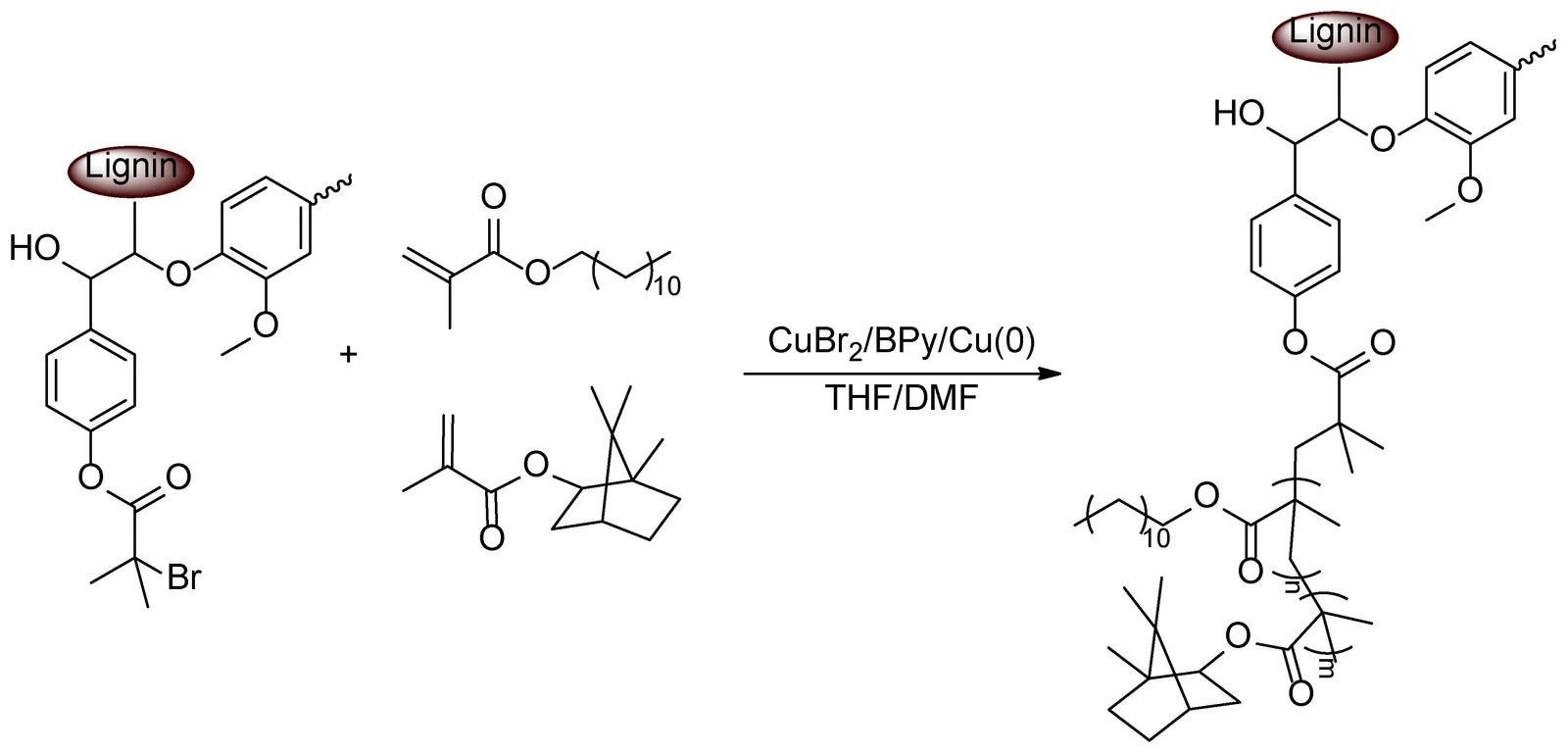
Examples of graft structure on lignin to build TPEs.

Along with synthetic ABA copolymers, modified bio-based macromolecules exhibiting superior mechanical properties over classic TPEs were also reported. For example, the rigid backbone of cellulose or lignin was linked to bio-based soft polymers. In particular, lignin was grafted with diblock copolymer chains having PLMA as the low *T*_g_ block and poly(isobornyl methacrylate) (PiBOMA) as a high *T*_g_ block. The PiBOMA proved to be an essential component in order to confer good mechanical properties (σ_b_ = 2.3 MPa, ε_b_ = 620%) and acceptable elastic recovery of the TPE in spite of having a *T*_g_ value close to room temperature (Figure 19) [146]. Another reported example of an LMA graft copolymer uses cellulose as the bio-based backbone. Three types of cellulose-based brush copolymers of tetrahydrofurfuryl methacrylate (THFMA) and LMA were investigated with random, dual, or block architectures. The authors found that the final properties could be tuned by the structure and copolymer composition, yielding materials with a good range of mechanical properties [147].

**Figure 20:**
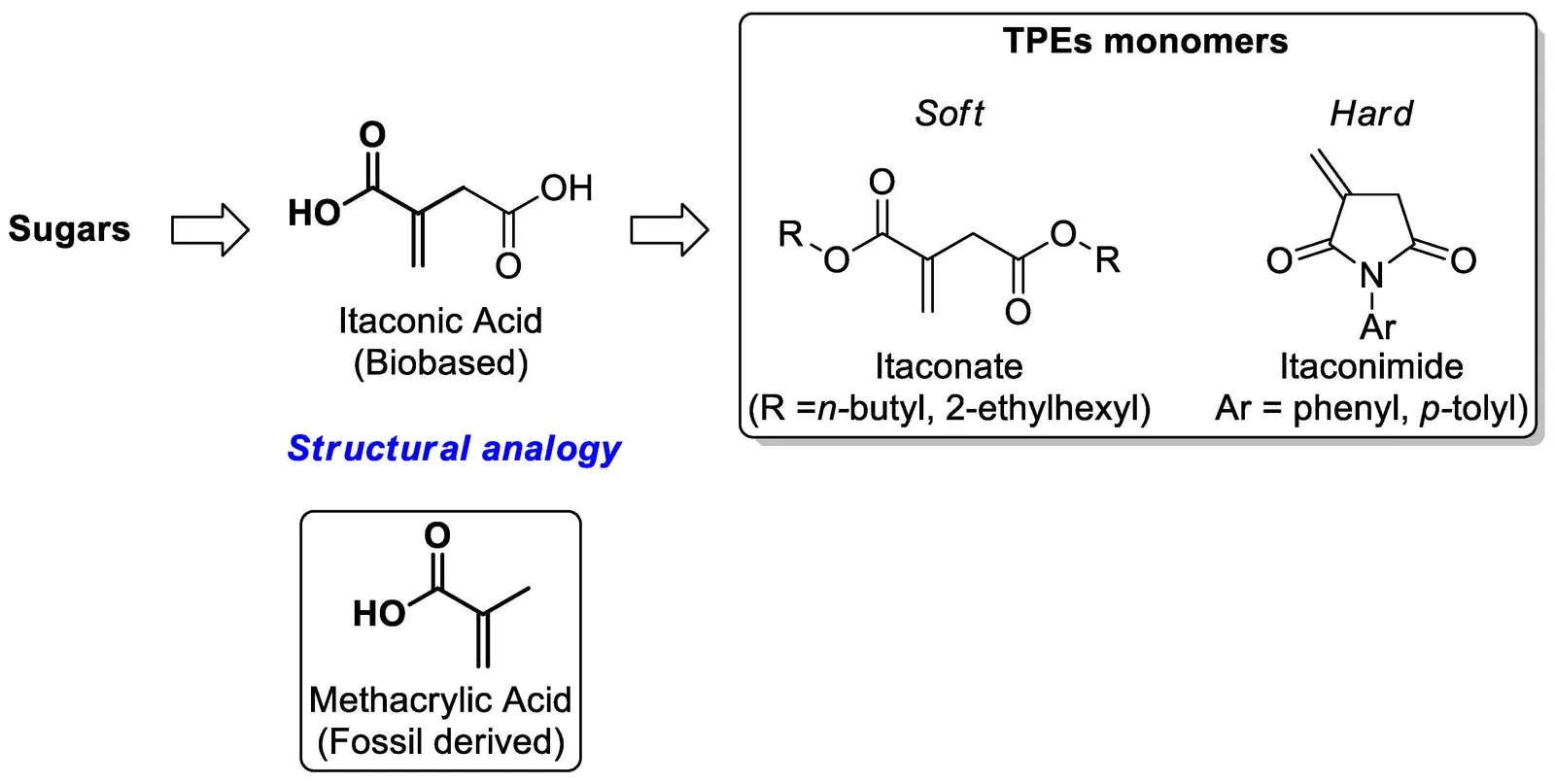
Biosourced itaconic acid-derived monomers for TPEs.

Plant sugars can also be transformed through bioprocessing into more functional building blocks, such as itaconic acid, whose structure presents similarities with that of methacrylic acid, hence representing a promising platform for methacrylic-like monomers (Figure 20) [148,149,150]. Indeed, depending on the nature of the side groups, the corresponding polymers can serve as either hard or soft blocks. Moreover, itaconic acid can be converted into its cyclic itaconic anhydride or imides, which are valuable bio-based hard monomers. In this scenario, Satho, Kamigaito, and coworkers reported on the sequential block RAFT copolymerisation of itaconates (dibutyl- and di-2-ethylhexyl itaconate) and itaconimides (phenyl- and *p*-tolyl itaconimides) as the soft and the hard monomers, respectively. Although the desired ABA structures were achieved, the mechanical properties of these materials were not reported [151].

Bio-based derived monomers potentially suitable as soft or glassy (*T*_g_ > 30 °C) building blocks for TPEs’ synthesis are listed in Table 2, whereas a summary of the bio-derived ABA systems is reported in Table 3.

## 6. TPEs Application

Due to their excellent overall mechanical performance and cost effectiveness, SBCs have become the industry standard for consumables such as ergonomic soft-touch grips for tools, toothbrushes, and writing instruments and are also used in the footwear industry (i.e., high-performance, shock-absorbing outsoles and flexible shoe components). In addition, SBCs offer promising prospects across diverse innovative applications, from wearable device sensors and next-generation fuel cell membranes to portable power sources for electric vehicles and shape memory polymers. Moreover, thanks to their biocompatibility, their potential extends to biomedical devices, including heart valves, urinary tract systems, coronary stent coatings, implants, and drug delivery networks [85]. The main SBC limitations are their poor-to-moderate environmental resistance (i.e., degradation upon exposure to UV light) and the need for formulations to ease the processability.

In contrast, acrylate-based TPEs exhibit high resistance to sunlight and outstanding optical transparency. Thanks to these features, such materials are employed in exterior components (weatherstripping, trim profiles, and sealing gaskets) in the automotive industry as well as in display technologies for highly transparent protective films, light guides, and optical-grade encapsulants. Because of their chemical inertness able to withstand intense sterilisation cycles, acrylate-based TPEs also find applications in medical and diagnostic fields. Nonetheless, because of their lower mechanical properties (due to the high molecular weight between entanglements) and higher production price compared to SBCs, the use of acrylate-based TPEs is still limited to premium/high products [152].

With respect to bio-based TPEs, in spite of the remarkable advances achieved during the last decade, along with the commercialisation of innovative, sometimes biodegradable, materials by multinational manufacturers, their applications remain limited [153,154,155]. ABA triblock bio-TPEs, based on polylactic acid (PLA) hard blocks and bio-derived poly(caprolactone) or poly(butyl acrylate) soft blocks, are employed in sustainable packaging and the medical sector [156]. It has to be noted that commercial products are mainly based on bio-TPUs and bio-TPOs, TPE families that are out of the scope of this review [157,158].

## 7. Conclusions and Future Perspective

TPEs represent a versatile class of polymeric materials that combine the elasticity of vulcanised rubbers with the processability and recyclability of thermoplastics through physical crosslinks generated by glassy or crystalline microdomains. In this review, we discussed ABA architectures, with particular focus on fossil-based SBCs, acrylic-based TPEs, and emerging bio-based systems. In conventional SBCs such as SBS and SIS, high-(*T*_g_) polystyrene (PS) end blocks (*T*_g_ ≈ 100 °C) form microphase-separated physical crosslinks around low-*T*_g_ polybutadiene or polyisoprene central blocks (*T*_g_ ≈ −90 and −56 °C, respectively), enabling elongations reaching 1000%; hydrogenated analogues such as SEBS, SEPS, and SIBS provide improved oxidative and UV stability, although hydrogenation may introduce crystallinity and alter mechanical behaviour. Acrylic-based ABA TPEs, exemplified by P(MMA-*b*-nBA-*b*-MMA), retain the same hard–soft–hard design and offer superior thermal and oxidative stability, optical transparency, oil resistance, weatherability, adhesion, printability, and abrasion resistance but generally exhibit lower mechanical performance than SBCs. This difference is partly associated with the substantially higher molecular weights between entanglements (*M*_e_) of acrylic soft blocks, ranging from approximately 11 kg/mol for poly(ethyl acrylate) and 28 kg/mol for poly(n-butyl acrylate) to as high as 60 kg/mol for poly(isooctyl acrylate), compared with approximately 1.7 kg/mol for polybutadiene and 6.1 kg/mol for polyisoprene.

The use of bio-derived monomers as partial or complete replacements for fossil-derived building blocks has further expanded the field, with the most promising systems using renewable monomers not simply as substitutes but as architectural building blocks to control (*T*_g_), phase separation, physical crosslink density, and entanglement characteristics. Particularly encouraging results have been obtained for terpene-derived TPEs; indeed, a β-myrcene-based ABA system containing α-methyl-p-methylstyrene hard blocks and a poly(β-myrcene) soft block achieved approximately 1200% elongation and 2.5 MPa tensile strength, with a service temperature up to 160 °C, while the menthide-based P(MBL-*b*-Mt-*b*-MBL) system reached similar elongation (1300%) and sensibly higher (13 MPa) tensile strength. The hydrogenated styrene-*b*-β-farnesene-*b*-styrene material SEPTON™ BIO further demonstrates the potential of renewable terpene-derived soft blocks, with mechanical performance reported to be comparable to or even better than that of oil-based hydrogenated SBCs. Bio-based acrylic systems provide a complementary approach, combining renewable high-*T*_g_ end blocks derived from sugar-, lignin-, and terpene-based monomers with flexible acrylic or lactone-derived middle blocks; for example, guaiacyl acrylate end blocks at contents below 5 mol% increased tensile strength by approximately 3–7-fold while retaining high elongation, whereas a guaiacyl methacrylate/hexalactone ABA system exhibited tensile strengths of approximately 1–2 MPa and elongations ranging from 900 to 3000%. Fully bio-based BetuMA/ET13 ABA copolymers further demonstrated that hard block identity and content strongly govern reinforcement, with moderate hard block fractions providing the best balance between strength and extensibility, while increasing the hard block content increased stiffness at the expense of elongation. It has to be noted that a punctual comparison between the performances of the materials might not be straightforward since mechanical testing is often non-standardised.

Overall, these results show that while fossil-based SBCs remain the benchmark for mechanical performance, acrylic-based TPEs offer advantages in thermal, oxidative, and functional versatility, and bio-based TPEs are increasingly capable of combining renewable feedstocks with competitive mechanical properties. The central challenge is therefore to reproduce the hierarchical molecular architecture responsible for efficient physical crosslinking, high entanglement density, and stable microphase separation while simultaneously reducing environmental impact. Future research should prioritise renewable soft blocks with mechanical characteristics closer to those of conventional diene-based elastomers, precise control over block molecular weight and dispersity, optimisation of hard/soft composition and microphase morphology, and scalable, greener synthesis and purification processes. In parallel, greater emphasis should be placed on non-food and waste-derived biomass feedstocks to avoid competition with food supply chains, together with comprehensive life-cycle assessment, durability, recyclability, and end-of-life recovery. The growing interest in this field is reflected in the global bio-based TPE market, valued at USD 118.15 million in 2024 and projected to reach USD 398.15 million by 2032, corresponding to a CAGR of 16.40% [159]. Ultimately, the development of sustainable high-performance TPEs will depend on integrating molecular-level architectural control with mechanical performance, processability, durability, and demonstrable environmental benefits, moving beyond simple fossil-resource substitution toward genuinely circular and sustainable elastomeric materials. In this regard, the development of advanced TPEs could be pursued by the introduction of novel crosslinking strategies aiming at extending their service temperature ranges. On this point, the incorporation of covalent adaptable networks and vitrimeric chemistry would represent a viable approach offering also a promising alternative to prevent mechanical degradation during multi-cycle reprocessing. These advancements must be complemented by atom-economical, solvent-free synthetic routes to minimise manufacturing footprints. Within this context, employing controlled radical polymerisation techniques proves particularly effective in precisely tailoring the ABA architecture. Ultimately, integrating comprehensive life-cycle assessments with standardised recycling protocols will be essential to transition advanced bio-based materials from laboratory-scale innovations into true, closed-loop industrial applications.

## Figures and Tables

**Table 1:** Comparison of the average molecular weight between chain entanglements of dienic and acrylic soft blocks [94].

Polymer	*M*_e_ (Da)
Poly(1,4-butadiene)	1700
Poly(isoprene)	6100
Poly(isobutylene)	8900
Poly(ethyl acrylate)	11,000
Poly(n-propyl acrylate)	16,000
Poly(n-butyl acrylate)	28,000
Poly(isooctyl acrylate)	60,000

**Table 2:** Bio-based monomers potentially suitable as soft (*T*_g_ < 20 °C) or glassy (*T*_g_ > 30 °C) building blocks for TPEs’ synthesis.

Bio Feedstock	Low-*T*_g_ Monomer	High-*T*_g_ Monomer
Plant sugars	Butyl lactate acrylate [134], Itaconic esters [151]	Isosorbide and glucose derivatives [123,124], itaconic amide [151]
Pine resin (turpentine and rosin)	β-Myrcene [112], β-Farnesene [116], Tetrahydrofurfuryl acrylate [97]	Dehydroabietic (meth)acrylates [113], β-Pinene-derived monomers [115,116]
Essential oils	Menthide [119], Tetrahydrogeraniol (meth)acrylates [127,128]	Thymol (meth)acrylates [132], menthol (meth)acrylates [122]
Fatty acids	n-alkyl (meth)acrylates [143,145], Lactones [138]	-
Lignin	-	Vanillin (meth)acrylates [134], syringol (meth)acrylates [133,135] guaiacol (meth)acrylates [138]

**Table 3:** Summary of the bio-based TPEs discussed in this review.

Bio Feedstock	Monomer	Role in ABA	Polymerisation Technique	TPE Type	Representative Mechanical Properties *^a^*
Terpene (pine resin)	β-Myrcene	Soft block	Living Anionic Polymerisation	P(AMMS-*b*-Myr-*b*-AMMS)	ε_b_ ≈ 1200% σ_b_ ≈ 2.5 MPa [117]
	β-Farnesene	Soft block	Living Anionic Polymerisation	Styrene-*b*-β-farnesene-*b*-Styrene	Lower toughness than SBS, improved processability [118]
	Menthide	Soft block	ATRP	P(MBL-*b*-Mt-*b*-MBL)	ε_b_ ≈ 1300% σ_b_ ≈ 13 MPa [119]
	Tetrahydrogeraniol (meth)acrylate	Soft block	RAFT	P(NPMA-*b*-THGMA-*b*-NPMA)	Good adhesion, promising mechanical properties [128]
Rosin derivative	Dehydroabietic ethyl methacrylate	Hard block	ATRP	P(DAEMA-*b*-nBA-*b*-DAEMA)	ε_b_ ≈ 240% σ_b_ ≈ 1.5 MPa [113]
	Nopinyl methacrylate	Hard block	RAFT	P(NPMA-*b*-THGMA-*b*-NPMA)	Phase-separated morphology; promising adhesion [127]
Phenols derived from lignin	Vanillin acrylate	Hard block	RAFT	P(VA-*b*-buLA-*b*-VA)	ε_b_ ≈ 250% σ_b_ ≈ 0.8 MPa [134]
	Guaiacyl acrylate	Hard block	RAFT	P(GA-*b*-MA-*b*-GA)	3–7-fold increase in tensile strength vs. PMA [137]
	Syringyl/Guaiacyl methacrylates	Hard block	RAFT	PSA triblocks	ε_b_ ≈ 500% σ_b_ < 1 MPa [135]
Plant sugars	Acetylated acrylic isosorbide	Hard block	RAFT	P(AAI-*b*-nBA-*b*-AAI)	ε_b_ ≈ up to 600% σ_b_ up to 6.5 MPa [123,124]
	Glucose acrylate	Hard block	RAFT	P(GATA-*b*-nBA-*b*-GATA)	ε_b_ ≈ 600% σ_b_ < 0.8 MPa [123,124]
	Butyl lactate acrylate	Soft block	RAFT	P(AAI-*b*-buLA-*b*-AAI)	ε_b_ ≈ 250% σ_b_ < 0.3 MPa [134]
	Itaconate derivatives	Soft block	RAFT	Itaconate triblocks	Not reported [151]
	Itaconimides	Hard block	RAFT	Itaconimide triblocks	Not reported [151]
Vegetable oils/fatty acids	Lauryl methacrylate	Soft block	ATRP	P(MMA-*b*-LMA-*b*-MMA)	ε_b_ ≈ 80–100% σ_b_ < 1–3 MPa [143,144]
		Soft block	RAFT	P(As-*b*-LMA-*b*-As)	ε_b_ ≈ 250% σ_b_ < 0.44 MPa [145]
	VISIOMER^®^ Terra C13	Soft block	RAFT	P(BetuMA-*b*-ET13-*b*-BetuMA)	Good elastomeric behaviour; properties dependent on hard block content [139]
Bio-based aromatics	*p*-Methoxystyrene	Hard block	RAFT	P(MOS-*b*-IBVE-*b*-MOS)	Good TPE behaviour [110]
	Dihydrofuran	Hard block	RAFT	P(DHF-*b*-IBVE-*b*-DHF)	Better recovery and strength than PMOS analogue [110]

*^a^* The reported mechanical properties were obtained under different testing conditions and should therefore be compared with caution.

## Data Availability

No new data were created or analysed in this study. Data sharing is not applicable to this article.
